# An Improved Continuous Control Set Model Predictive Current Control Algorithm for Permanent Magnet Synchronous Motor

**DOI:** 10.3390/s26144593

**Published:** 2026-07-20

**Authors:** Wenfei Yu, Dejie Bo, Yanbin Han, Jia Han, Yufa Duan, Xin Wang, Ping Lin

**Affiliations:** 1 School of Control Science and Engineering, Dalian University of Technology, Dalian 116024, China; 2 AECC Shenyang Engine Research Institute, Shenyang 110015, China

**Keywords:** permanent magnet synchronous motor, continuous control set model predictive control, harmonic suppression, linear–nonlinear switching extended state observer

## Abstract

Aiming at the issue of poor robustness in continuous control set model predictive current control (CCS-MPCC) algorithm, a class of model predictive controllers based on linear–nonlinear switching extended state observers has been designed. Theoretical derivations demonstrate the structural equivalence between the extended state observer-based deadbeat model predictive current controller and the active disturbance rejection control (ADRC) and the errors introduced into the MPC algorithm’s predictive output by parameters such as motor resistance, flux linkage, and inductance have been calculated. Subsequently, a linear–nonlinear state observer was employed to detect disturbances in the system caused by parameter deviations and the observed disturbance values were fed back into the current loop controller in real time, thereby improving the prediction accuracy of the CCS-MPCC algorithm. Finally, the control algorithm was verified on a 5.5 kW experimental platform of permanent magnet synchronous motor (PMSM). The experimental results show that when the model parameters and actual parameters have deviations, the model predictive current control algorithm based on the linear–nonlinear switching extended state observer has a better speed response effect, and can reduce the harmonic content of the original method by up to 1.03%, 1.25%, and 1.12% under three operating conditions.

## 1. Introduction

In recent years, with the rapid advancement of microprocessor computing speeds, the advantages of model predictive control (MPC) in the field of motor control have become increasingly apparent. MPC is an advanced control technology developed based on optimal control theory. Its primary feature is to predict the future state of the motor based on discrete models of the inverter and motor, as well as the motor’s current state. It then compares the predicted values with predefined evaluation criteria to select the optimal voltage vector to apply to the motor [1]. Compared to traditional control strategies, MPC offers faster dynamic response, stronger online optimization capabilities, a simpler algorithmic structure, and the ability to introduce additional constraints based on control objectives [2]. The use of MPC controllers in electric aircraft to ensure rapid dynamic response of the powertrain system is both feasible and practical.

MPC can be classified into continuous control set model predictive control (CCS-MPC) and finite control set model predictive control (FCS-MPC) based on the type of optimization problem. MPC can also be classified based on the controller’s position into model predictive speed control (MPSC) and model predictive current control (MPCC). MPSC performs predictive control based on the motor’s mechanical motion equations, while MPCC performs predictive control based on the motor’s voltage equations. Based on the discrete or continuous state of the control set, MPSC and MPCC can be classified into finite control sets and continuous control sets. A finite control set is characterized by the controller directly outputting switching signals without undergoing space vector pulse width modulation (SVPWM) tuning; therefore, it can only output eight basic voltage vectors; control signals generated by continuous control sets undergo SVPWM or sinusoidal pulse width modulation (SPWM), theoretically enabling the output of voltage vectors in any direction within a two-phase stationary coordinate system, resulting in a magnetic field that more closely approximates a circular shape.

In finite control set model predictive speed control (FCS-MPSC), the inverter’s switching states form a discrete control set, allowing the motor to be driven directly without pulse width modulation. There are many algorithms based on the FCS-MPSC concept, including defining current attraction regions [3], switching between steady-state and transient modes to generate torque-related current components [4,5,6], specifying torque trajectories via a sliding manifold [7], adjusting the equivalent speed tracking error [8], and calculating the reference voltage based on the deadbeat control concept [9,10,11]. Due to the lack of SVPWM modulation, FCS-MPSC typically fails to achieve satisfactory steady state performance.

Unlike FCS-MPSC, continuous control set model predictive speed control (CCS-MPSC) employs an SVPWM modulation algorithm, and its control set is continuous. This results in significantly improved steady state performance. In existing literature, the prediction horizon of CCS-MPSC is generally short. Liu implemented a non-cascaded CCS-MPSC by using a full-order PMSM model and linearizing it to decouple speed and current [12].

Valencia-Palomo proposed a multi-parameter predictive control method based on Laguerre functions (mpLOMPC), which, using input sequence reparameterization, reduces the average number of mp-QP partitions by approximately 50% while expanding the feasible region without compromising performance [13]. A journal article further implemented mpLOMPC in a PLC using the IEC 61131-3 standard and verified the algorithm’s real-time performance and engineering feasibility through motor speed control experiments [14,15].

The prediction horizon of CCS-MPSC is typically short, which may lead to stability issues. To address this, Liu employed Laguerre functions to extend the prediction horizon of CCS-MPSC. Simulation and experimental results demonstrated that this strategy improves system stability and steady state performance while maintaining high dynamic performance [16]. On the other hand, Carlet implemented a cascaded control structure, which consists of a speed control loop and a current control loop [17].

Continuous control set model predictive current control (CCS-MPCC) obtains the optimal continuous voltage vector through the current loop cost function or by solving a quadratic programming problem. Subsequently, modulation algorithms such as SVPWM are used to convert the continuous voltage vector into a pulse width modulation (PWM) signal, which is applied to the inverter to drive the motor. Currently, the most widely used CCS-MPCC is deadbeat predictive current control (DPCC). DPCC ensures that the actual current tracks its reference value without delay and has attracted widespread attention from researchers due to its excellent dynamic performance, higher control accuracy, and easily designed controller structure. Zhang proposed a simple DPCC method with improved parameter robustness, enhancing the system’s robustness [18]. Favato proposed a fast and accurate implicit CCS-MPC for the current control loop of a synchronous motor drive with input constraints, ensuring optimal current performance of the system and applying it to real-world industrial scenarios [19].

The primary distinction between FCS-MPCC and CCS-MPCC lies in whether SVPWM technology is employed in the system. FCS-MPCC performs current prediction using the motor’s discrete voltage equations and outputs switching signals to the inverter, thereby achieving rapid system response. Since the algorithm does not employ signal modulation techniques, FCS-MPCC offers a more intuitive theoretical framework and provides a more convenient and rapid approach to handling nonlinear constraints and multivariable control problems. Building upon FCS-MPCC, Zhang proposed an improved FCS-MPCC method based on an incremental model, which effectively eliminates the impact of parameter mismatch on control performance and reduces the parameter sensitivity of the MPCC method [20].

When electric aircraft operate for extended periods, motor parameters typically change. Since MPC controllers rely heavily on the model parameters of the controlled system, this causes a deviation between the motor parameters in the predictive model and the actual parameters, further leading to a decline in the control performance of the entire control system. Therefore, the main focus of this paper is to address the sensitivity of model predictive control to the model parameters of the controlled system by improving the model predictive controller, and to apply the improved robust model predictive controller to PMSM to enhance the operational performance of aircraft ducted fan motors.

Although FCS-MPC algorithms can easily handle multi objective control with constraints, they suffer from high current ripple and large variations in switching frequency due to their simple modulation strategies. Discrete voltage vectors cause torque ripples during switching, generating electromagnetic interference that significantly reduces motor lifespan. Consequently, CCS-MPC strategies are preferred in practical engineering applications. Among CCS-MPC algorithms, the deadbeat current control algorithm has attracted attention from researchers both domestically and internationally due to its fast dynamic response, excellent static tracking performance, and low computational complexity [21]. Li proposed a simple solution for adjusting the parameters of nonlinear extended state observers. They also conducted a quantitative analysis and comparative study of linear active disturbance rejection control (LADRC) and nonlinear active disturbance rejection control (NADRC), proposed a LADRC/NADRC switching control scheme, and verified the feasibility of the switching active disturbance rejection controller scheme using a ball-and-beam platform [22]. Building on Li’s work, Lin further investigated a class of linear–nonlinear switching active disturbance rejection control strategies. Using a 5.5 kW high-speed towed test platform, Lin demonstrated that this switching algorithm can enhance the disturbance rejection capabilities of PMSM speed and current controllers [23].

Hao proposed a Linear/nonlinear ADRC switching control (SADRC) that switches between L/NLADRC based on error and disturbance values, using a linear combination to ensure a smooth transition, with parameters tuned using the reference bandwidth method. Experiments demonstrated that it outperforms LADRC, NLADRC, and PI in all performance metrics [24]. Qu designed an SADRC based on a new switching function, where the parameter $\theta_{2}$ adjusts the switching point for both ESO and SEF. Theoretical analysis confirmed convergence and stability, and experiments showed that it combines the fast response of LADRC with the high accuracy of NLADRC, resulting in optimal overall performance [25]. Chen proposed a cascaded linear–nonlinear ADRC, with a front-end LESO to stabilize large disturbances and a back-end NLESO to correct residuals, using a linear error feedback law and proving stability via the Routh criterion. Experiments showed that its velocity/position errors outperformed those of PLL, LADRC, and cascaded LADRC [26].

The method proposed in this paper is a typical robust deadbeat predictive control strategy based on an extended state observer. Its core lies in the use of a linear–nonlinear switching extended state observer (LNS-ESO) to perform real-time estimation and feedforward compensation of the total system disturbance. Unlike existing approaches that use a single linear or nonlinear observer, this method adaptively switches between the LESO and NESO operating modes based on the magnitude of the observation error, balancing high-precision estimation under small errors with stability under large disturbances, thereby enhancing the robustness of the control system across a wide range of operating conditions. At the same time, the prediction model is still constructed based on the motor’s physical parameters, and the observer is used solely to compensate for residual disturbances; there is no need to reconstruct the system model, thereby preserving the advantages of the DPCC structure—namely, its simplicity and intuitive parameter tuning. This switching mechanism effectively integrates the respective advantages of linear and nonlinear approaches at the observer design level, which is the key factor distinguishing it from other hybrid methods.

## 2. Problem Description and Analysis

Although single-vector model predictive current control (SVMPCC) is widely used in various industrial applications due to its ease of implementation and low computational complexity, the algorithm is limited by discrete sets, making it difficult to guarantee control accuracy in low-speed or high-speed control scenarios. SVMPCC can only output a voltage vector of a fixed direction within each control cycle $T_{s}$, making it difficult for the current $i_{q}$ to track the setpoint current $i_{q}^{*}$ after one cycle.

**Figure 1:**
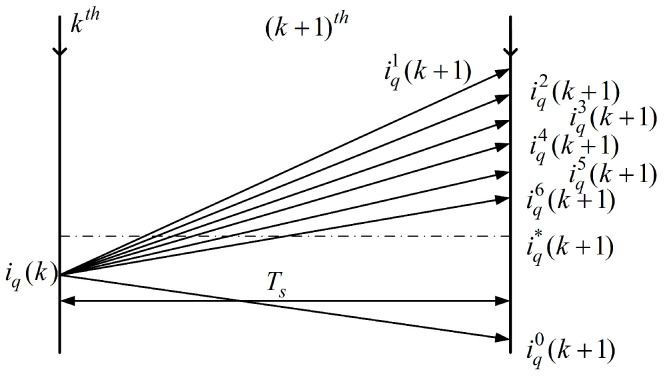
Computational schematic of the single-vector algorithm.

As shown in Figure 1, taking the motor’s state $i_{q}\left(k\right)$ at time *k* as an example, the current loop control period is $T_{s}$. Due to the algorithmic limitations of SVMPCC, there are only seven fixed voltage vector sets: $i_{q}^{0}\left(k\right)$, $i_{q}^{1}\left(k\right)$, $i_{q}^{2}\left(k\right)$, $i_{q}^{3}\left(k\right)$, $i_{q}^{4}\left(k\right)$, $i_{q}^{5}\left(k\right)$ and $i_{q}^{6}\left(k\right)$ (the zero-voltage vector and six basic voltage vectors). This makes it difficult for $i_{q}\left(k+1\right)$ to track the setpoint value $i_{q}^{*}\left(k\right)$ within a single cycle, and the resulting current error $e_{q}\left(k+1\right)$ is the root cause of motor system instability. The current error for current control using the single-vector model at time *k* is given by

(1) $$e_{q}\left(k+1\right)=i_{q}^{6}\left(k+1\right)-i_{q}^{*}\left(k\right)$$

**Figure 2:**
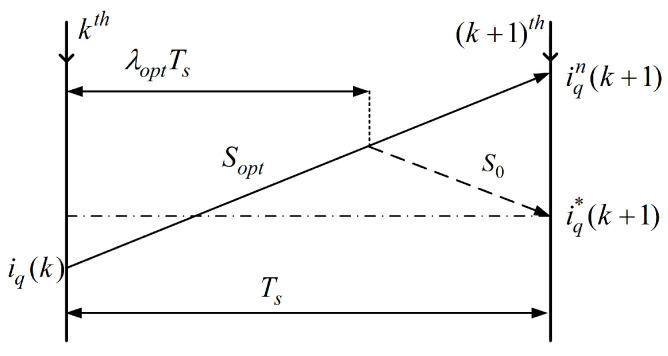
Schematic of the duty-cycle algorithm.

To address this issue in SVMPCC, researchers have proposed duty-cycle model predictive control based on the deadbeat control concept. Unlike SVMPCC, in duty-cycle model predictive control, the optimal voltage vector selected based on the optimal cost function is active only for a portion of the sampling period, denoted as $\lambda_{opt}\;\cdot \;T_{s}$ ($\lambda_{opt}\in \left[0,1\right]$). The duration $\lambda_{opt}\;\cdot \;T_{s}$ during which the voltage vector is active is calculated by the cost function, while the remaining time $\left(1-\lambda_{opt}\right)\;\cdot \;T_{s}$ is occupied by the zero-voltage vector. Taking the q-axis current deadbeat as an example for analysis, as shown in Figure 2.

During a control cycle, the slope $s_{opt}$ and $s_{0}$ are controlled respectively through the control optimal voltage vector and the weight $\Gamma_{opt}$ of the zero voltage vector action time so that $i_{q}\left(k+1\right)$ reaches the setpoint value $i_{q}^{*}\left(k\right)$ at the next time step, as shown in Equation (2):

(2) $$i_{q}\left(k+1\right)=i_{q}\left(k\right)+s_{opt}t_{opt}+s_{0}\left(T_{s}-t_{opt}\right)=i_{q}^{*}\left(k\right)$$

where $t_{opt}$ is the duration of the optimal voltage vector selected at that time, $s_{opt}$ is the slope of $i_{q}$ during the application of the optimal voltage vector, and $s_{0}$ is the slope of $i_{q}$ during the application of the zero-voltage vector. Based on the current prediction formula, the calculation formulas for slopes $s_{0}$ and $s_{opt}$ are

(3) $$s_{0}=\frac{di_{q}}{dt}{|}_{0}=\frac{1}{L}\left[-Ri_{q}\left(k\right)-\omega_{e}Li_{d}\left(k\right)-\omega_{e}\psi_{f}\right]$$

(4) $$s_{opt}=\frac{di_{q}}{dt}{|}_{opt}=s_{0}+\frac{u_{q}{|}_{opt}}{L}$$

where $u_{q}{|}_{opt}$ is the optimal voltage vector at time *k*. Substituting Equations (3) and (4) into Equation (2) yields the duty-cycle $\lambda_{opt}$ of the optimal voltage vector:

(5) $$\lambda_{opt}=\frac{t_{opt}}{T_{s}}=\frac{i_{q}^{*}-i_{q}\left(k\right)-s_{0}T_{s}}{T_{s}\left(s_{opt}-s_{0}\right)}\;\;\;\lambda_{opt}\in \left[0,1\right]$$

Finally, the zero vector and the selected optimal voltage vector, along with their respective application times $\left(1-\lambda_{opt}\right)\;\cdot \;T_{s}$ and $\lambda_{opt}\;\cdot \;T_{s}$, are applied to the inverter to drive the motor.

Duty-cycle model predictive current control addresses the issue that SVMPCC cannot precisely track the current setpoint within a single control cycle, thereby reducing torque ripple in the system. However, duty-cycle model predictive control also has its own issues: since a zero vector is introduced in every control cycle, the computational load of duty-cycle model predictive control is equivalent to that of dual vector model predictive control. This substantial computational load can easily cause control board processing delays in motor drive systems, thereby affecting the dynamic and steady-state performance of the motor system. To enable the controller to accurately track the current setpoint within a single control cycle while reducing the system’s computational load, deadbeat prediction current control was developed to address this issue.

Whether using SVMPCC or duty-cycle model predictive control, the slope of $i_{q}$ remains constant for any setpoint voltage during a single control cycle. Although duty-cycle model predictive control can track $i_{q}^{*}$ without static error, it still requires time-weighted allocation. This is due to the limitations of finite control set model predictive control algorithms. The FCS-MPCC controller drives the inverter by directly outputting switching signals, resulting in the output of only a finite voltage vector per cycle. A finite voltage vector not only reduces tracking accuracy relative to the setpoint but also significantly increases the computational load of the algorithm. Deadbeat predictive current control generates switching signals to drive the inverter through SVPWM modulation. This allows DPCC to output voltage vectors in any direction in the two-phase stationary coordinate system within the same control cycle.

**Figure 3:**
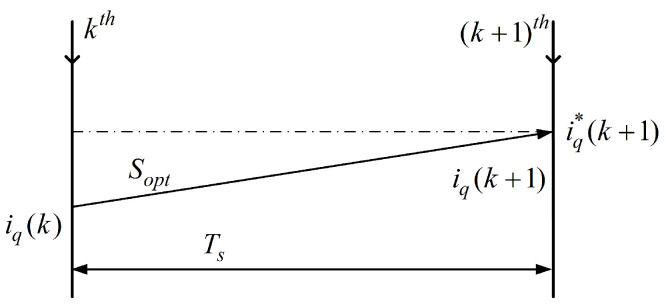
Schematic of the deadbeat algorithm.

As shown in Figure 3, based on the setpoint current $i_{q}^{*}$ at time *k*, DPCC allows the slope of the current $i_{q}\left(k\right)$ to take on any value, and the slope $s_{opt}$ is no longer limited to the 8 basic voltage vectors. To ensure that the current tracks the setpoint value within a single operating cycle, the slope $s_{opt}$ is set to

(6) $$s_{opt}=\frac{i_{q}^{*}\left(k\right)-i_{q}\left(k\right)}{T_{s}}$$

DPCC algorithm calculates the current slope using Equation (6). The fundamental principle of DPCC is to predict the motor stator current at the next time based on discrete mathematical models of the motor and inverter, and then generate corresponding switching signals via the SVPWM algorithm, thereby achieving precise control of the stator current within a short time. It features a fast current loop dynamic response, low harmonics, and effective control of motor torque, resulting in excellent speed servo performance. Since DPCC allows the generation of voltage vectors in any direction, the actual controller output more closely approximates the theoretical optimal solution.

By transforming the motor voltage equation in the two-phase rotating coordinate system and performing forward Euler discretization, we obtain Equation (7):

(7) $$\left\{\begin{array}{c} u_{d}\left(k\right)=\frac{L}{T}\;\cdot \;i_{d}\left(k+1\right)+\left(R-\frac{L}{T}\right)\;\cdot \;i_{d}\left(k\right)-L\;\cdot \;\omega_{e}\;\cdot \;i_{q}\left(k\right)\\ u_{q}\left(k\right)=\frac{L}{T}\;\cdot \;i_{q}\left(k+1\right)+\left(R-\frac{L}{T}\right)\;\cdot \;i_{q}\left(k\right)+L\;\cdot \;\omega_{e}\;\cdot \;i_{d}\left(k\right)+\omega_{e}\;\cdot \;\psi_{f} \end{array}\right.$$

where $u_{d}\left(k\right)$ and $u_{q}\left(k\right)$ are the voltages that need to be applied at time *k*; while $i_{d}\left(k\right)$ and $i_{q}\left(k\right)$ represent the predicted current values at time *k* for different basic voltage vectors; $i_{d}\left(k+1\right)$ and $i_{q}\left(k+1\right)$ are the predicted values of the current at time k+1; $\omega_{e}$ is the motor’s electrical angular velocity; resistance *R*, inductance *L*, and flux linkage $\psi_{f}$ are the three basic motor parameters in the current prediction model. Rearranging Equation (7) yields Equation (8):

(8) $$\left\{\begin{array}{c} u_{d}\left(k\right)=L\frac{i_{d}^{*}\left(k\right)-i_{d}\left(k\right)}{T_{s}}+Ri_{d}\left(k\right)-L\omega_{e}\left(k\right)i_{q}\left(k\right)\\ u_{q}\left(k\right)=L\frac{i_{q}^{*}\left(k\right)-i_{q}\left(k\right)}{T_{s}}+Ri_{q}\left(k\right)+L\omega_{e}\left(k\right)i_{d}\left(k\right)+\omega_{e}\left(k\right)\psi_{f} \end{array}\right.$$

According to Equation (8), the current loop reference value $i_{q}^{*}\left(k\right)$ at time *k* is used as the predicted value for the algorithm at time k+1, and the corresponding $u_{d}\left(k\right)$ and $u_{q}\left(k\right)$ are output. At each sampling time, the outputs required for the next cycle are calculated to ensure that the controlled variable reaches the desired setpoint within a finite number of time steps. This control technique is commonly used for current control in power electronics and motor control systems, enabling the feedback current to track the current reference value $i_{q}^{*}\left(k\right)$ within one to two control cycles.

Assuming the sampling time is sufficiently short, it can be assumed that the reference currents $i_{d}^{*}$ and $i_{q}^{*}$ in the d-q rotating coordinate system remain virtually unchanged between two adjacent time intervals. This characteristic of the reference current is expressed by the following relationship:

(9) $$\left\{\begin{array}{c} i_{d}^{*}\left(k+2\right)\approx i_{d}^{*}\left(k+1\right)\\ i_{q}^{*}\left(k+2\right)\approx i_{q}^{*}\left(k+1\right)\\ i_{d}^{*}\left(k+1\right)\approx i_{d}^{*}\left(k\right)\\ i_{q}^{*}\left(k+1\right)\approx i_{q}^{*}\left(k\right) \end{array}\right.$$

Additionally, due to limitations in the controller’s processing speed, DPCC requires one-step delay compensation. First, the current values $i_{d}^{*}\left(k+1\right)$ and $i_{q}^{*}\left(k+1\right)$ at time k+1 are predicted using Equation (1), and then, based on Equation (9), the formula for the deadbeat predicted current control with one-step delay compensation is derived:

(10) $$\left\{\begin{array}{c} u_{d}\left(k+1\right)=L\frac{i_{d}^{*}\left(k\right)-i_{d}^{p}\left(k+1\right)}{T_{s}}+Ri_{d}^{p}\left(k+1\right)-L\omega_{e}\left(k+1\right)i_{q}^{p}\left(k+1\right)\\ u_{q}\left(k+1\right)=L\frac{i_{q}^{*}\left(k\right)-i_{q}^{p}\left(k+1\right)}{T_{s}}+Ri_{q}^{p}\left(k+1\right)+L\omega_{e}\left(k+1\right)i_{d}^{p}\left(k+1\right)+\omega_{e}\left(k+1\right)\psi_{f} \end{array}\right.$$

where $u_{d}\left(k+1\right)$ and $u_{q}\left(k+1\right)$ represent the basic voltage vectors to be output at time k+1, these voltage values are calculated using the cost function; $i_{d}^{p}\left(k+1\right)$ and $i_{q}^{p}\left(k+1\right)$ denote the one-step delay compensation predicted currents on the d-axis and q-axis, respectively, at time k+1 when the system is subject to disturbances.

**Figure 4:**
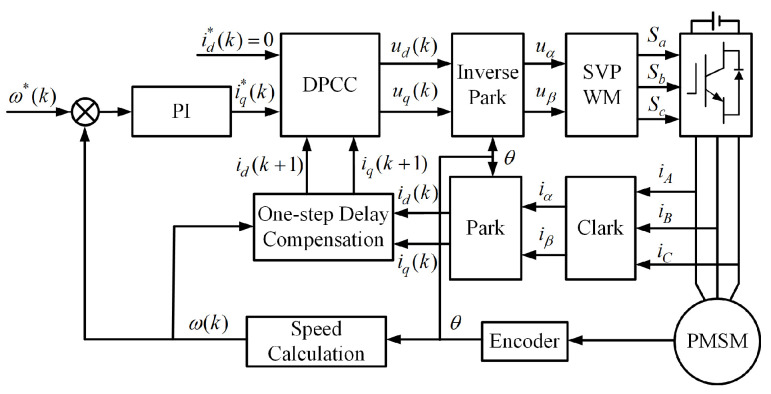
Structure of DPCC.

The system block diagram of the DPCC is shown in Figure 4. Due to the use of the SVPWM algorithm, DPCC ensures precise voltage tracking while requiring only one-sixth of the computational load of SVMPCC. The system’s dynamic response is faster than that of SVMPCC; therefore, it is gradually replacing traditional FCS-MPC methods in high-performance drive systems (such as in the electric vehicle sector and precision servo applications).

Similar to SVMPCC, the DPCC algorithm also heavily relies on the current prediction model of PMSM. Therefore, if there is a mismatch between the motor model parameters in the algorithm and those of the actual motor, such as errors in the parameters of resistance *R*, inductance *L*, and flux linkage $\psi_{f}$, which can all lead to inaccurate model predictions, thereby affecting the system’s control performance. Therefore, we continue to investigate the error between the predicted and actual outputs of the current loop, obtained through theoretical calculations, when there is a mismatch between the model parameters and the actual parameters in the deadbeat prediction current control algorithm.

Based on Equation (8) and considering the parameter disturbances present in the system, the DPCC prediction model can be expressed as follows:

(11) $$\left\{\begin{array}{c} u_{d}\left(k\right)=\left(L+\Delta L\right)\frac{i_{d}^{*}\left(k\right)-i_{d}\left(k\right)}{T_{s}}+\left(R+\Delta R\right)i_{d}\left(k\right)-\left(L+\Delta L\right)\omega_{e}\left(k\right)i_{q}\left(k\right)\\ u_{q}\left(k\right)=\left(L+\Delta L\right)\frac{i_{q}^{*}\left(k\right)-i_{q}\left(k\right)}{T_{s}}+\left(R+\Delta R\right)i_{q}\left(k\right)+\left(L+\Delta L\right)\omega_{e}\left(k\right)i_{d}\left(k\right)+\omega_{e}\left(k\right)\left(\psi_{f}+\Delta \psi_{f}\right) \end{array}\right.$$

where ΔR is the deviation between the actual resistance and the model resistance; ΔL is the deviation between the actual inductance and the model inductance; and $\Delta \psi_{f}$ is the deviation between the actual rotor flux linkage and the model flux linkage. By taking the difference between Equation (8) and Equation (11), the theoretical error in the current loop at time *k* when the model parameters do not match the actual parameters can be calculated as

(12) $$\left\{\begin{array}{c} E_{ud}\left(k\right)=\frac{\Delta L}{T}\;\cdot \;e_{id}\left(k+1\right)+\Delta R\;\cdot \;i_{d}\left(k\right)-\Delta L\;\cdot \;\omega_{e}\;\cdot \;i_{q}\left(k\right)\\ E_{uq}\left(k\right)=\frac{\Delta L}{T}\;\cdot \;e_{iq}\left(k+1\right)+\Delta R\;\cdot \;i_{q}\left(k\right)+\Delta L\;\cdot \;\omega_{e}\;\cdot \;i_{d}\left(k\right)+\Delta \psi_{f}\;\cdot \;\omega_{e} \end{array}\right.$$

where $E_{ud}\left(k\right)$ and $E_{uq}\left(k\right)$ represent the current loop output errors on the d-axis and q-axis, respectively, at time *k* in the DPCC algorithm; $e_{id}\left(k+1\right)$ and $e_{iq}\left(k+1\right)$ are the differences between the current setpoints and feedback values on the d-axis and q-axis, as shown in Equation (13):

(13) $$\left\{\begin{array}{c} e_{id}\left(k+1\right)=i_{d}^{*}\left(k\right)-i_{d}\left(k+1\right)\\ e_{iq}\left(k+1\right)=i_{q}^{*}\left(k\right)-i_{q}\left(k+1\right) \end{array}\right.$$

Mathematical derivation of the error Equation (12) reveals a nonlinear coupling relationship between the inductance parameters and the d-q axis current prediction error. This nonlinear characteristic makes it difficult to effectively correct the predicted current error using a feedforward compensation strategy with fixed gain when there is a deviation between the motor model parameters and the actual parameters. It is particularly worth noting that the rotor flux linkage parameters only affect the q-axis current prediction equation; errors in these parameters will directly cause systematic deviations in q-axis current tracking, and the magnitude of the deviation is linearly positively correlated with the flux linkage error.

In practical applications, DPCC control typically requires one-step delay compensation. Therefore, when parameter errors exist in the system, Equation (14) is expressed as follows:

(14) $$\left\{\begin{array}{c} u_{d}\left(k+1\right)=\left(L+\Delta L\right)\frac{i_{d}^{*}\left(k\right)-i_{d}^{p}\left(k+1\right)}{T_{s}}+\left(R+\Delta R\right)i_{d}^{p}\left(k+1\right)-\left(L+\Delta L\right)\omega_{e}\left(k+1\right)i_{q}^{p}\left(k+1\right)\\ u_{q}\left(k+1\right)=\left(L+\Delta L\right)\frac{i_{q}^{*}\left(k\right)-i_{q}^{p}\left(k+1\right)}{T_{s}}+\left(R+\Delta R\right)i_{q}^{p}\left(k+1\right)+\left(L+\Delta L\right)\omega_{e}\left(k+1\right)i_{d}^{p}\left(k+1\right)+\omega_{e}\left(k+1\right)\left(\psi_{f}+\Delta \psi_{f}\right) \end{array}\right.$$

where $i_{d}^{p}\left(k+1\right)$ and $i_{q}^{p}\left(k+1\right)$ denote the one-step delay compensation predicted currents on the d-axis and q-axis, respectively, at time k+1 when the system is subject to disturbances.

Subtracting Equation (13) from Equation (10) yields the current prediction error formula incorporating one-step delay compensation:

(15) $$\left\{\begin{array}{c} E_{ud}\left(k+1\right)=L\frac{E_{id}\left(k+1\right)}{T_{s}}+\Delta L\frac{e_{id}\left(k+1\right)-E_{id}\left(k+1\right)}{T_{s}}+RE_{id}\left(k+1\right)+\Delta Ri_{d}^{p}\left(k+1\right)-L\omega_{e}\left(k+1\right)E_{iq}\left(k+1\right)-\Delta L\omega_{e}\left(k+1\right)i_{q}^{p}\left(k+1\right)\\ E_{uq}\left(k+1\right)=L\frac{E_{iq}\left(k+1\right)}{T_{s}}+\Delta L\frac{e_{iq}\left(k+1\right)-E_{iq}\left(k+1\right)}{T_{s}}+RE_{iq}\left(k+1\right)+\Delta Ri_{q}^{p}\left(k+1\right)+L\omega_{e}\left(k+1\right)E_{id}\left(k+1\right)+\Delta L\omega_{e}\left(k+1\right)i_{d}^{p}\left(k+1\right) \end{array}\right.$$

where $E_{id}\left(k+1\right)$ and $E_{iq}\left(k+1\right)$ represent the current prediction errors at time k+1; $i_{d}^{p}\left(k+1\right)$ and $i_{q}^{p}\left(k+1\right)$ represent the one-step predicted currents at time k+1.

From Equations (14) and (15), it can be seen that when one-step delay compensation is present in the system, the prediction current error and current loop output voltage error caused by mismatch are further amplified. Consequently, the one-step delay compensation intended to improve control system performance actually further degrades the system’s control performance. Since the current loop output error in the DPCC includes part of the predicted current error from the SVMPCC, the DPCC exhibits sensitivity to motor parameters such as resistance *R*, inductance *L*, and flux linkage $\psi_{f}$ that is similar to that of the SVMPCC. A mismatch in any one of the parameters—resistance *R*, inductance *L*, or flux linkage $\psi_{f}$—will result in errors in the predicted current. However, the inductance error ΔL forms a complex nonlinear coupling term with rotational speed and voltage, and its impact on control accuracy is far greater than that of the resistance error ΔR and the flux linkage error $\Delta \psi_{f}$. Therefore, in subsequent research, to ensure the effectiveness of the experiments, experiments involving inductance mismatch were prioritized. Consequently, parameter mismatch has a significant impact on the DPCC algorithm, substantially degrading the system’s control performance. To address this issue, it is necessary to propose an improved algorithm to reduce the DPCC’s parameter sensitivity and enhance the robustness of the control system.

## 3. Design of an Improved CCS-MPCC

Based on the analysis in the previous section, by reorganizing Equations (7) through (15), we obtain the discrete motor equations under the DPCC algorithm that include parameter errors, expressed as follows:

(16) $$\left\{\begin{array}{c} u_{d}\left(k\right)=L\frac{i_{d}^{*}\left(k\right)-i_{d}\left(k\right)}{T_{s}}+Ri_{d}\left(k\right)-L\omega_{e}\left(k\right)i_{q}\left(k\right)+E_{ud}\\ u_{q}\left(k\right)=L\frac{i_{q}^{*}\left(k\right)-i_{q}\left(k\right)}{T_{s}}+Ri_{q}\left(k\right)+L\omega_{e}\left(k\right)i_{d}\left(k\right)+\omega_{e}\left(k\right)\psi_{f}+E_{uq} \end{array}\right.$$

By considering the system’s partial state variables and parameter errors as the total disturbances $f_{ud}$, $f_{uq}$, and adjusting the structure of Equation (16), it is transformed into the following form:

(17) $$\left\{\begin{array}{c} u_{d}\left(k\right)=L\;\cdot \;\frac{i_{d}^{*}\left(k\right)-i_{d}\left(k\right)}{T}-L\;\cdot \;f_{ud}\left(k\right)\\ u_{q}\left(k\right)=L\;\cdot \;\frac{i_{q}^{*}\left(k\right)-i_{q}\left(k\right)}{T}-L\;\cdot \;f_{uq}\left(k\right) \end{array}\right.$$

where $f_{ud}\left(k\right)$ and $f_{uq}\left(k\right)$ represent the total system disturbances present on the d-q axis, respectively. A conclusion can be drawn here: observing Equation (17), if the portion of the DPCC system excluding the error term is treated as the total disturbance and this total disturbance is compensated for in the DPCC controller via extended state observer (ESO), the ESO-based DPCC system can be regarded as an active disturbance rejection controller with fixed parameters in the feedback control law. The two controllers are structurally equivalent. Based on this conclusion, when designing the ADRC controller, we can treat it as an ESO-based robust DPCC controller. In this case, all parameters in the feedback control law can be tuned according to Equation (17), which significantly reduces the parameter tuning effort for the ADRC controller.

Compared to NESO, LESO is more conducive to theoretical research, and its performance does not degrade significantly as the amplitude or rate of change of the total disturbance increases. However, LESO’s tracking performance is inferior to that of NESO, and because the gain is constant, initial state errors may cause the LESO system to exhibit peak phenomena. In contrast, NESO offers higher tracking accuracy, stronger disturbance rejection, and faster response times, among other advantages. Furthermore, due to the presence of nonlinear mechanisms, NESO is relatively insensitive to initial state errors. However, the stability and performance of NESO systems are difficult to analyze, and traditional frequency theory is not readily applicable; when the amplitude or derivative of the total disturbance increases beyond a certain threshold, performance degrades sharply.

Since both LESO and NESO systems have their own advantages and disadvantages, combining the strengths of LESO and NESO to leverage their respective advantages can further enhance the system’s disturbance rejection capability. Based on the research by Li and Lin [23,24], this chapter seamlessly integrates LESO and NESO through observation errors and applies a linear–nonlinear switching active disturbance rejection controller to the design of the DPCC algorithm for PMSM, thereby enhancing the robustness of the model predictive control system. This observer, which integrates the advantages of LESO and NESO, is referred to as the linear–nonlinear switching extended state observer (LNS-ESO). This switching control scheme combines the advantages of LESO and NESO. By using LNS-ESO to observe the DPCC system instead of NESO, the robustness of the DPCC system is further enhanced through the higher observation accuracy and more stable control performance of LNS-ESO.

The switching scheme for the LNS-ESO is as follows: the LNS-ESO automatically switches between LESO and NESO based on the relationship between the $z_{1}$ term and the errors $e_{d}$ and $e_{q}$ relative to the observed currents $i_{d}$ and $i_{q}$. The parameters $\delta_{1}$ and $\delta_{2}$ of the LNS-ESO are defined, and the values of the errors $|e_{d}|$ and $|e_{q}|$ are divided into three intervals: $\left[0,\delta_{1}\right]$, $\left[\delta_{1},\delta_{2}\right]$, and $\left[\delta_{2},+\infty \right]$. When the observation error *e* is less than $\delta_{2}$, the observer adopts the NESO control strategy; when the observation error *e* is greater than $\delta_{2}$, the observer adopts the LESO control strategy. For the DPCC algorithm, a second-order LNS-ESO is employed. To verify the effectiveness of the linear–nonlinear switching observer, this section adopts two linear–nonlinear switching observers, named LNS1 and LNS2, respectively. The structure of LNS1 is shown in Equation (18):

(18) $$\left\{\begin{array}{c} {\dot{z}}_{1}=z_{2}-\beta_{11}\;\cdot \;e_{1}+b_{0}\;\cdot \;u\\ {\dot{z}}_{2}=-F_{2}\;\cdot \;fal\left(e_{1},\alpha_{2},\delta_{1},\delta_{2}\right) \end{array}\right.$$

where $z_{1}$ is the current estimate, $z_{2}$ is the estimate of the total disturbance of the DPCC system, and $F_{2}\;\cdot \;fal\left(e_{1},\alpha_{2},\delta_{1},\delta_{2}\right)$ is the linear–nonlinear switching function, which can employ discontinuous switching. Its structure is shown in Equation (19):

(19) $$F_{2}\;\cdot \;fal\left(e_{1},\alpha_{2},\delta_{1},\delta_{2}\right)=\left\{\begin{array}{c} \beta_{23}\;\cdot \;\frac{e_{1}}{\delta_{1}^{1-\alpha_{2}}}\;\;\;\;\;\;\left|e_{1}\right|\leq \delta_{1}\\ \beta_{22}\;\cdot \;{\left|e_{1}\right|}^{\alpha_{2}}\;\cdot \;\mathrm{sign}\left(e_{1}\right)\;\;\delta_{1}<\left|e_{1}\right|\leq \delta_{2}\\ \beta_{21}\;\cdot \;e_{1}\;\;\;\;\;\;\;\left|e_{1}\right|>\delta_{2} \end{array}\right.$$

where $\beta_{11}$, $\beta_{21}$, $\beta_{22}$, and $\beta_{23}$ are the gain coefficients of the linear–nonlinear switching observer LNS1. The stability of LNS1 is proved in Appendix A.1.

The structure of LNS2 is shown in Equation (20):

(20) $$\left\{\begin{array}{c} {\dot{z}}_{1}=z_{2}-F_{1}\;\cdot \;fal\left(e_{1},\alpha_{1},\delta_{1},\delta_{2}\right)+b_{0}\;\cdot \;u\\ {\dot{z}}_{2}=-F_{2}\;\cdot \;fal\left(e_{1},\alpha_{2},\delta_{1},\delta_{2}\right) \end{array}\right.$$

where $z_{1}$ is the estimated current, $z_{2}$ is the estimated total disturbance of the DPCC system; $F_{i}\;\cdot \;fal\left(e_{1},\alpha_{i},\delta_{1},\delta_{2}\right)$ is the linear–nonlinear switching function, which may employ discontinuous switching. Its structure is shown in Equation (21):

(21) $$F_{i}\;\cdot \;fal\left(e_{1},\alpha_{i},\delta_{1},\delta_{2}\right)=\left\{\begin{array}{c} \beta_{i3}^{\prime }\;\cdot \;\frac{e_{1}}{\delta_{1}^{1-\alpha_{i}}}\;\;\;\;\;\;\left|e_{1}\right|\leq \delta_{1}\\ \beta_{i2}^{\prime }\;\cdot \;{\left|e_{1}\right|}^{\alpha_{i}}\;\cdot \;\mathrm{sign}\left(e_{1}\right)\;\;\delta_{1}<\left|e_{1}\right|\leq \delta_{2}\\ \beta_{i1}^{\prime }\;\cdot \;e_{1}\;\;\;\;\;\;\;\left|e_{1}\right|>\delta_{2} \end{array}\right.$$

where $\beta_{11}^{\prime }$, $\beta_{12}^{\prime }$, $\beta_{13}^{\prime }$, $\beta_{21}^{\prime }$, $\beta_{22}^{\prime }$, and $\beta_{23}^{\prime }$ are the gain coefficients of the linear–nonlinear switching observer LNS2. The stability of LNS2 is proved in Appendix A.2.

**Figure 5:**
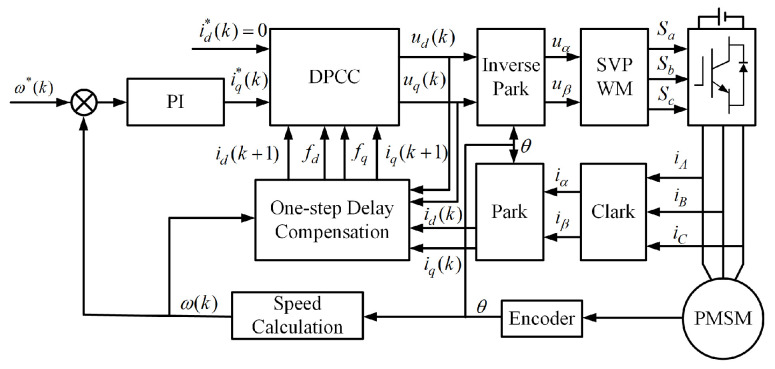
The structure of robust DPCC.

The boundary parameter $\delta_{2}$ between LESO and NESO can be determined through simulation, experimentation, or both. During simulation, disturbances are introduced into the nominal model, and $\delta_{2}$ is repeatedly adjusted until its performance exceeds that of both NESO and LESO. Alternatively, during experimentation, disturbances are introduced into the actual model, and $\delta_{2}$ is repeatedly adjusted until its performance exceeds that of both NESO and LESO. The improved algorithm framework is shown in Figure 5.

## 4. Experimental Validation and Results Analysis

**Figure 6:**
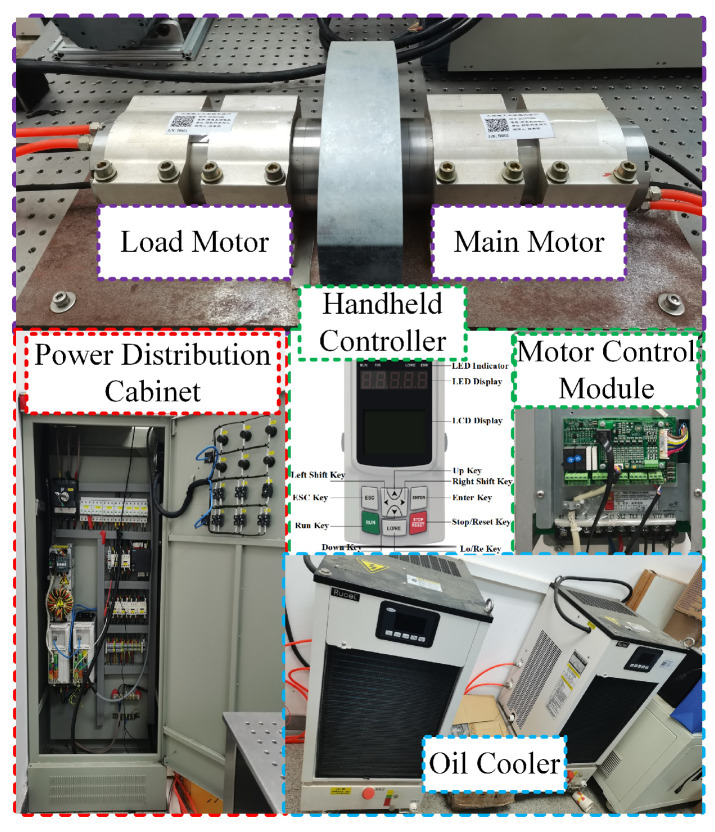
The 5.5 kW experimental platform of PMSM.

The 5.5 kW experimental platform of PMSM used in this study is shown in Figure 6. The platform consists of two PMSMs: the main motor on the right and the load motor on the left. The load motor is used to apply load disturbances to the main motor. The detailed motor parameters are listed in Table 1. Additionally, the system includes control and drive boards for the main and load motors, a power distribution cabinet, two oil coolers, a host computer for data acquisition and processing, and a handheld controller.

**Figure 7:**
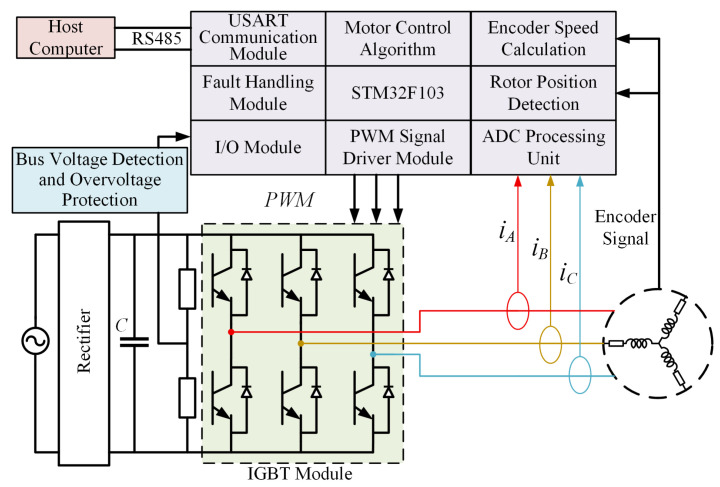
Hardware structure of PMSM.

The hardware architecture of the permanent magnet synchronous motor control system is shown in Figure 7. The control board serves as the core of PMSM drive system, performing programming and motor control on an STM32F103VC chip based on the ARM Cortex-M3 core. Its computing speed is 72 MHz and supports single-cycle multiplication and hardware division instructions; It is equipped with two 12-bit analog-to-digital converters (ADCs), supporting 16-channel sampling with a conversion rate of 1 μs; it also features an integrated temperature sensor; It features a 7-channel DMA controller, supporting efficient data transfer between peripherals and memory; communication interfaces include 3 USARTs, 2 I2Cs, 3 SPIs, 1 CAN 2.0B, and 1 USB 2.0 full speed interface; it also includes 2 advanced control timers (supporting PWM output), 4 general purpose timers, and 2 watchdog timers.

The control board is programmed in C. It is responsible for acquiring stator current and performing A/D conversion; receiving differential signals from an incremental encoder to obtain rotor position and speed information; acquiring DC bus voltage for system protection; communicating with the host computer via an RS485 serial port to enable status monitoring and command exchange; and outputting PWM signals to drive the inverter circuit.

The drive circuit uses an AS500 vector-controlled inverter, which rectifies 380 VAC to a 540 VDC bus voltage $U_{DC}$, which is then inverted by an IGBT inverter into three-phase sinusoidal pulses to drive PMSM. The IGBT switching devices receive PWM signals from the control board to turn on and off, and by adjusting the switching frequency, the voltage amplitude and frequency output to PMSM are modified.

**Figure 8:**
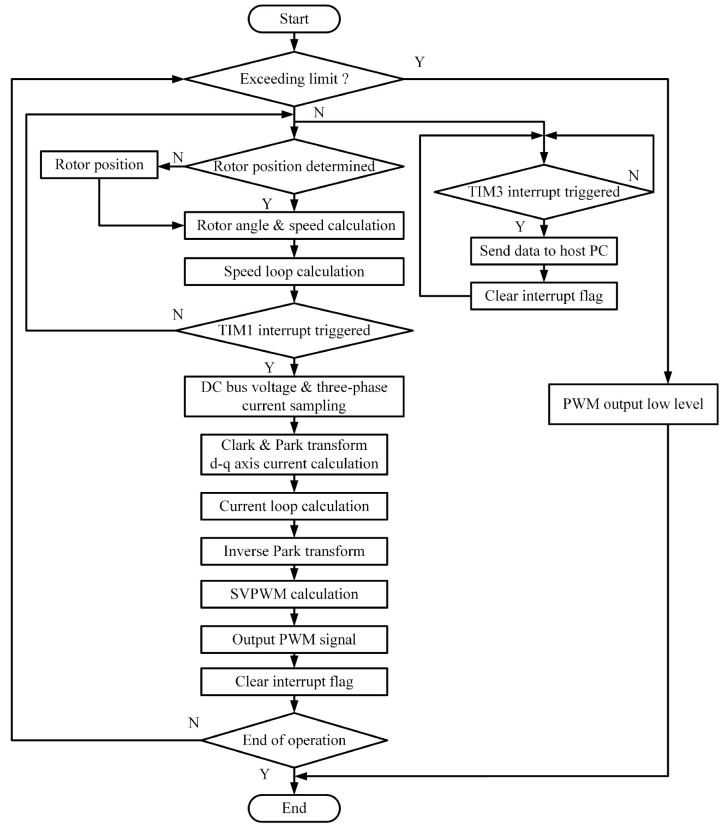
Flowchart of the control program.

The control program flow is shown in Figure 8. Before each execution of the control program, the system must check the circuit’s current status; if the current exceeds the limit, the PWM signal output is forcibly stopped to protect the circuit. The main program runs in a loop using a ‘while’ function, with speed sampling and speed loop calculations performed within the main program at a 1-millisecond cycle. Timer 1 is used to generate periodic TIM1 interrupts, with an execution cycle of 1/6 ms. Within the TIM1 interrupt service routine, the following modules are executed: DC bus voltage and three-phase current sampling; Clark and Park transformations of the three-phase currents; current loop calculation; inverse Park transformation; SVPWM calculation and output PWM signal. Upon completion of one cycle of the interrupt service routine, the interrupt flag is cleared, and the function exits to await the next interrupt. Simultaneously, Timer 3 generates a 5 ms periodic interrupt, which is used to transmit relevant experimental data to the host computer.

**Figure 9:**
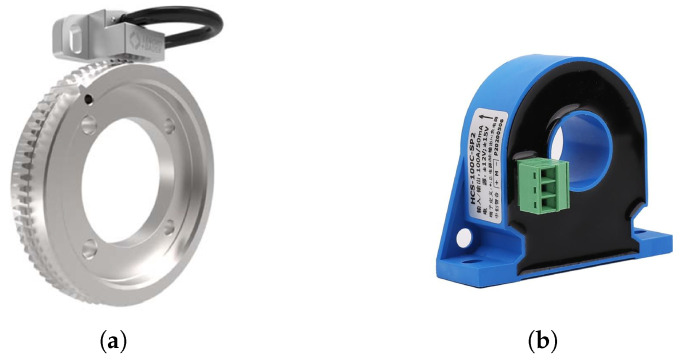
Incremental differential encoder and Hall current sensor: (**a**) Incremental differential encoder. (**b**) Hall current sensor.

The detection of rotor position and speed employs an incremental differential encoder based on a magnetoresistive sensor, whose hardware structure is shown in Figure 9a. This encoder consists of a miniature encoder and an 80-line precision measuring gear; the gear is mounted directly on the rotor shaft to enable non-contact measurement. The miniature encoder contains a built-in magnetic field that changes as the gear rotates. By detecting these changes in the magnetic field, the encoder outputs differential signals A+, A−, B+, B−, Z+, and Z−, thereby obtaining information on the rotor’s rotation direction, speed, and position.

Hall-effect current sensors are used to measure three-phase stator current, as shown in Figure 9b. These sensors operate based on the Hall effect: the magnetic field generated around a current-carrying conductor is proportional to the current flowing through it. The Hall device detects the magnitude of this magnetic field and outputs a corresponding voltage signal; the output voltage *U* is linearly related to the current $i_{s}$ being measured, i.e., $i_{s}\propto B\propto U$. By measuring the voltage value, the magnitude of the stator current can be calculated. The sensors have a rated current of 100 A, a measurement range of 0–150 A, a turns ratio of 1:1000, and a rated output of 100 mA corresponding to a primary rated current of 100 A. Its rise time is less than 1 μs, the current change rate $\frac{di}{dt}$ exceeds 50 A/μs, and the frequency range is 0–100 kHz. The current loop control of the software platform operates at a sampling period of $T_{S}=$ 166.67 μs, corresponding to a sampling frequency of 6 kHz; the speed loop sampling period is longer than that of the current loop, at 1 ms, corresponding to a sampling frequency of 1 kHz.

To verify the effectiveness of the algorithm described in the previous section, experimental validation was conducted on a 5.5 kW permanent magnet synchronous motor-to-motor physical test platform. The controller parameters were selected based on the optimal performance of each observer system. Next, the plan is to deeply couple the PINN loss function design with the threshold optimization process, build a data-driven adaptive adjustment mechanism, use neural networks to sense the system state in real time, and optimize $\delta_{1}$, $\delta_{2}$ online, with further validation of its effectiveness through an extended experimental platform later.

The parameters for LESO are $\beta_{1}=1260$, $\beta_{2}=53.3$;

The parameters for NESO are $\beta_{1}=1260$, $\beta_{2}=53.3$, $a_{1}=0.889$, $a_{2}=0.5$, $\delta_{1}=4$;

The parameters for LNS1 are $\beta_{1}=1260$, $\beta_{21}=40$, $\beta_{22}=40$, $\beta_{23}=180$, $a_{2}=0.5$, $\delta_{1}=4$, $\delta_{2}=9$;

The parameters of LNS2 are $\beta_{11}=90$, $\beta_{12}=100$, $\beta_{13}=260$, $\beta_{21}=40$, $\beta_{22}=90$, $\beta_{23}=180$, $a_{1}=0.889$, $a_{2}=0.5$, $\delta_{1}=4$, $\delta_{2}=9$.

The experimental setup was as follows: a step command of 3000 r/min was applied to the speed loop. After the motor started under no-load conditions and reached steady-state operation, a load disturbance of 0.5 Nm was applied, and the load was removed after 4 s. Observe the dynamic and steady-state response characteristics of the motor’s speed, current, and torque throughout the process, and perform a quantitative fast Fourier transform (FFT) spectral analysis of the stator winding current, with a focus on the total harmonic distortion (THD) of the system after the load disturbance is applied.

(22) $$THD=\frac{\sqrt{\sum_{h=2}^{N}I_{h}^{2}}}{I_{1}}\times 100\%$$

where $I_{1}$ is the root mean square (RMS) value of the fundamental current, $I_{h}$ is the RMS value of the *h*th harmonic current, and *N* is the highest harmonic order.

The model inductances of the motor are defined as $L_{1}$, $L_{2}$, and $L_{3}$, representing changes in motor inductance under different operating conditions, respectively. The actual motor inductance is $L_{s}$. Based on simple loading and unloading experiments, the actual motor inductance is set as follows: Condition I: $L_{1}=L_{s}$; Condition II: $L_{2}=0.5L_{s}$; and Condition III: $L_{3}=0.3L_{s}$, the control performance of the DPCC algorithm, the LESO based improved model predictive control, the NESO based improved model predictive control, the LNSESO1 based improved model predictive control, and the LNSESO2 based improved model predictive control is observed. For the sake of clarity, this section refers to the four improved methods as the improved LESO method, the improved NESO method, the improved LNS1 method, and the improved LNS2 method, respectively.

**Figure 10:**
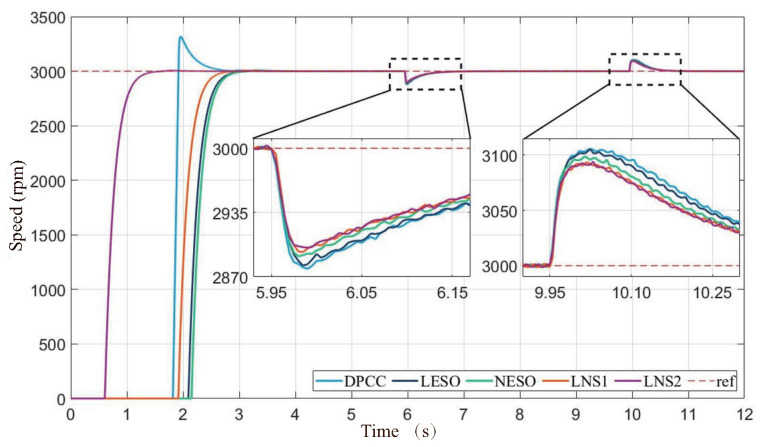
Comparison curve of motor speed response in Condition I.

The speed responses of the DPCC algorithm, the improved LESO method, the improved NESO method, the improved LNS1 method and the improved LNS2 method under Condition I are shown in Figure 10.

Under Condition I, as shown in the speed response in Figure 10, when the model parameters match the actual motor parameters, the DPCC algorithm system shows a speed overshoot of 3326.09 rpm during no-load operation, whereas the speed responses of the four improved methods show no overshoot. After applying a load disturbance and then unloading, the modified LNS2 method exhibits the smallest speed drop and speed rise values. The speed drop and rise values of the modified LNS1 method are greater than those of the modified LNS2 method; the modified NESO method is greater than the modified LNS1 method; the modified LESO method is greater than the modified NESO method; and the DPCC method exhibits the largest speed drop and rise values. Therefore, in terms of speed performance under Condition I, the modified LNS2 method exhibits the strongest disturbance rejection capability, followed by the modified LNS1 method. The modified LNS1 method outperforms the modified NESO method, which in turn outperforms the modified LESO method, while the DPCC method yields the poorest results.

During the startup phase, the settling times of the five algorithms, ranked from best to worst, were LNS2 (0.57 s), DPCC (1.81 s), LNS1 (1.92 s), LESO (2.09 s), and NESO (2.15 s), indicating that different observer structures have a significant impact on startup dynamics. After applying the load, the speed fluctuations of the five algorithms, ranked from best to worst, were LNS2 (2899.08 rpm), LNS1 (2894.46 rpm), NESO (2890.53 rpm), LESO (2880.95 rpm), and DPCC (2878.04 rpm). After the load was removed, the speed fluctuations of the five algorithms, ranked from best to worst, were LNS1 (3092.87 rpm), LNS2 (3093.94 rpm), NESO (3098.49 rpm), LESO (3105.3 rpm), and DPCC (3105.86 rpm). Both LNS2 and LNS1 ranked among the top performers overall. It should be noted that this paper focuses on comparing the noise-rejection performance of the various algorithms; the underlying mechanisms of the startup response and optimization strategies are beyond the scope of this study, and related research will be explored in greater depth in future work.

**Figure 11:**
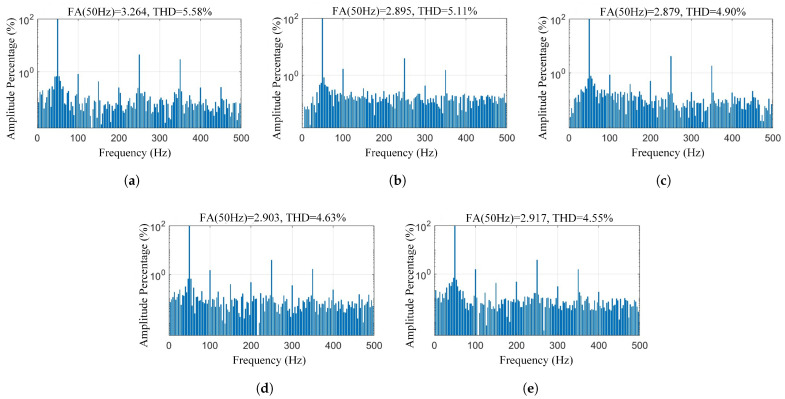
Stator current FFT analysis of five systems in Condition I: (**a**) DPCC. (**b**) LESO. (**c**) NESO. (**d**) LNS1. (**e**) LNS2.

To visually analyze the algorithms’ effectiveness in improving system current, THD analysis was performed on the motor stator current after applying disturbances, and the system’s current response was evaluated using the THD value. The THD analysis results of the stator current for the five methods under Condition I are shown in Figure 11, FA represents the fundamental amplitude.

Analysis of Table 2 and Figure 11 reveal that under Condition I, the DPCC method exhibits the highest harmonic content and the poorest response performance for stator current; the improved LESO method reduces harmonic content by approximately 0.47% compared to the DPCC method; the improved NESO method reduces harmonic content by approximately 0.68%; and the improved LNS1 method reduces harmonic content by approximately 0.95%; the improved LNS2 method yields the lowest THD value and reduces the harmonic content of the DPCC method by approximately 1.03%. Under Condition I, the improved LNS2 method demonstrates the strongest disturbance suppression capability, while the DPCC method exhibits the weakest.

**Figure 12:**
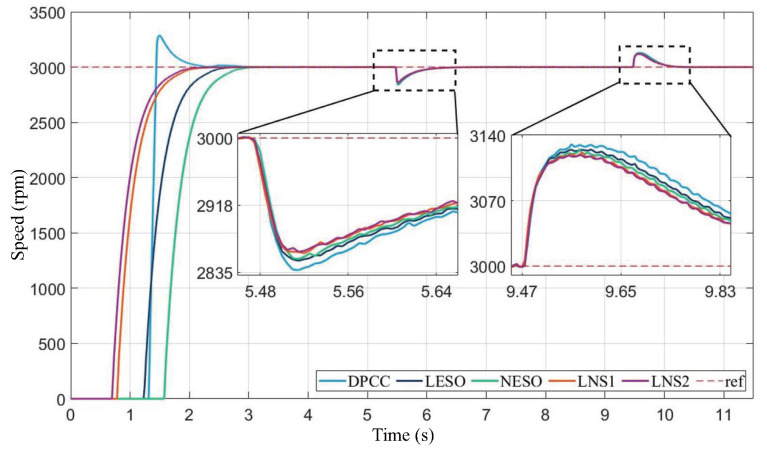
Comparison curve of motor speed response in Condition II.

**Figure 13:**
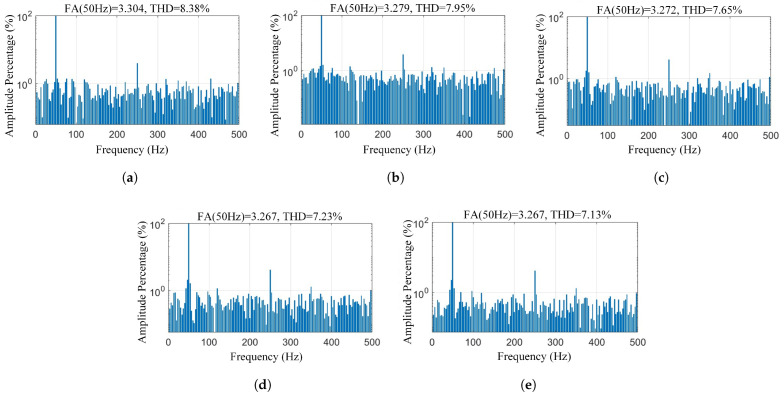
Stator current FFT analysis of five systems in Condition II: (**a**) DPCC. (**b**) LESO. (**c**) NESO. (**d**) LNS1. (**e**) LNS2.

Figure 12 shows the speed and response characteristics of the five methods under Condition II. As shown in the speed response in Figure 12, When the model parameters are set to 0.5 times the actual motor parameters, the DPCC algorithm system shows a speed overshoot of 3291.67 rpm during no-load operation. while the speed responses of the four improved methods show no overshoot. After applying a load disturbance and then unloading, the improved LNS2 method exhibited the smallest speed drop and speed rise values. The speed drop and rise values for the improved LNS1 method were slightly larger than those of the improved LNS2 method; the improved NESO method was larger than the improved LNS1 method; the improved LESO method was larger than the improved NESO method; and the DPCC method exhibited the largest speed drop and rise values. Therefore, it can be concluded that under Condition II, the improved LNS2 method exhibits the best speed performance, followed by the improved LNS1 method, while the DPCC method has the worst speed response. The FFT analysis of the stator currents for the five methods under the loading conditions of Condition II is shown in Figure 13; the three-phase currents were measured using current clamps.

During the startup phase, the settling times of the five algorithms, ranked from best to worst, were: LNS2 (0.69 s), LNS1 (0.88 s), LESO (1.23 s), DPCC (1.32 s), and NESO (1.58 s), indicating that different observer structures have a significant impact on startup dynamics. After applying a load, the LNS2 method exhibited the best performance in terms of rotational speed fluctuations, with a minimum of 2860.27 rpm; the DPCC method performed the worst, with a minimum of 2837.92 rpm. The LNS2 method continued to demonstrate stable and efficient performance.

Analysis of the second column in Table 2 and Figure 13 show that under Condition II, the DPCC method still has the highest harmonic content and the poorest stator current response. The improved LESO method reduces the harmonic content by approximately 0.43% compared to the DPCC method; the improved NESO method reduces it by approximately 0.73%; and the improved LNS1 method reduces it by approximately 1.15%; The improved LNS2 method yields the lowest THD value, reducing the harmonic content of the DPCC method by approximately 1.25%. Under Condition II, the improved LNS2 method still demonstrates the strongest disturbance suppression capability, while the DPCC method exhibits the weakest. Comparing the first column of Table 2 with the second column reveals that, as the error between the predicted and actual inductance parameters increases, the THD value of the stator current in the DPCC method rises, and the harmonic content increases. However, the THD values of the four improved methods remain lower than those of the DPCC algorithm; therefore, the improved methods can suppress current distortion and reduce harmonic content to a certain extent.

**Figure 14:**
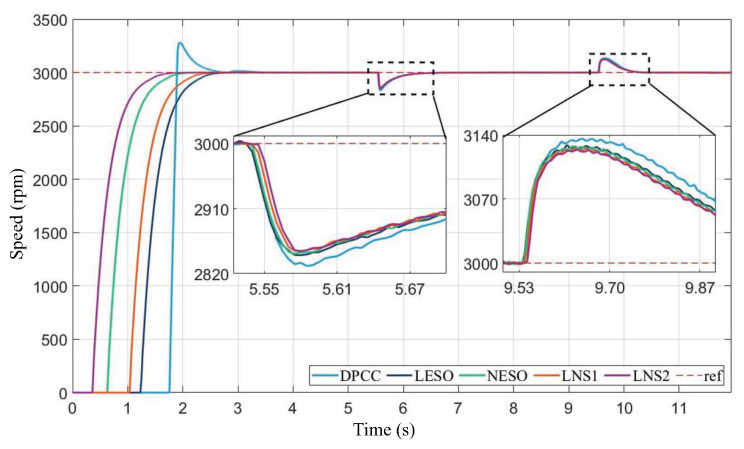
Comparison curve of motor speed response in Condition III.

To further verify the impact of changes in model inductance on the system, experiments were conducted under Condition III. The speed responses of the five methods under Condition III are shown in Figure 14. As can be seen from the speed responses in Figure 14, when the model parameters are set to 0.3 times the actual motor parameters, the system’s dynamic response performance deteriorates significantly. During no-load operation, the DPCC algorithm system shows a speed overshoot of 3286.81 rpm, while the speed responses of the other four methods remain free of overshoot. After applying a load disturbance and then unloading, the speed responses of the four improved methods showed little difference. The improved LNS2 method exhibited the smallest speed drop and rise values, followed by the improved LNS1 method, which was slightly larger than the improved LNS2 method. The improved NESO method was slightly larger than the improved LNS1 method, and the improved LESO method was slightly larger than the improved NESO method; however, the DPCC method still exhibited the largest speed drop and rise values. Therefore, under the extreme conditions of Condition III, the speed response of the improved methods remains superior to that of the DPCC method.

During the startup phase, the settling times of the five algorithms, ranked from best to worst, were: LNS2 (0.36 s), NESO (0.63 s), LNS1 (1.03 s), LESO (1.23 s), and DPCC (1.76 s), indicating that different observer structures have a significant impact on startup dynamics. After applying a load, the LNS2 method exhibited the best performance in terms of rotational speed fluctuations, with a minimum of 2850.68 rpm; the DPCC method performed the worst, with a minimum of 2830.28 rpm. The LNS2 method continued to demonstrate stable and efficient performance.

**Figure 15:**
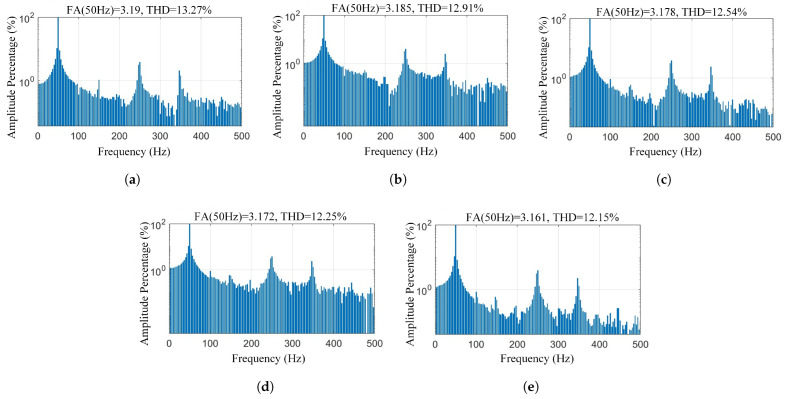
Stator current FFT analysis of five systems in Condition III: (**a**) DPCC. (**b**) LESO. (**c**) NESO. (**d**) LNS1. (**e**) LNS2.

Furthermore, across all three operating conditions, the improved LNS2 method performed best among the four improved systems, followed by the improved LNS1 method; the improved NESO method performed worse than the improved LNS1 method, and the improved LESO method performed the worst. The THD analysis results of the stator current for the five methods under the loading conditions of Condition III are shown in Figure 15.

Under Condition III, the improved LNS1, improved LNS2, improved NESO, and improved LESO methods can reduce the THD value of the DPCC method and decrease the degree of current distortion, in Figure 15. Even when significant disturbances are present in the system, the improved methods can still reduce the system’s harmonic content. The improved LESO method reduces the harmonic content of the DPCC method by approximately 0.36%; the improved NESO method reduces harmonic content by approximately 0.73%; the improved LNS1 method reduces harmonic content by approximately 1.02%; the improved LNS2 method yields the lowest THD value, reducing harmonic content by approximately 1.12%. Under extreme operating conditions, the improved LNS2 method still demonstrates the strongest disturbance suppression capability, while the DPCC method exhibits the weakest disturbance suppression capability. Comparing the first, second, and third columns of Table 2 reveals that as system parameter errors gradually increase, the THD value of the stator current in the DPCC method continues to rise, and the harmonic content increases significantly. In contrast, the improved methods can suppress the degree of current distortion in the system to a certain extent and reduce the system’s harmonic content. The current performance of the improved LNS1 and improved LNS2 methods under the three operating conditions is superior to that of the improved NESO and improved LESO methods.

A comparison of the motor speed response curves under Conditions I, II, and III reveals that, compared to the DPCC algorithm, the improved methods proposed in this paper—based on a linear–nonlinear switching observer—optimize system overshoot, enhance speed dynamic response and stability, and deliver the best speed response performance across different operating conditions.

Table 2 summarizes the THD analysis results of the DPCC algorithm, the improved LESO method, the improved NESO method, the improved LNS1 method, and the improved LNS2 method under the three operating conditions. Table 3 compares the improvements in system robustness achieved by the four improved methods under the three operating conditions.

As shown in Table 3, the improved methods based on linear–nonlinear switching observers adopted in this paper can reduce the oscillation of the system’s stator current when model parameters do not match actual parameters, lower the system’s harmonic content, reduce the sensitivity of the DPCC algorithm to model parameters, improve the system’s steady-state performance, and enhance system robustness. Furthermore, the performance improvements achieved by the improved LNS1 and improved LNS2 methods are superior to those of the improved NESO and improved LESO methods. The improved LNS1 method reduces the harmonic content of the original DPCC method by 0.95%, 1.15%, and 1.02% under the three operating conditions, respectively; the improved LNS2 method reduces the harmonic content of the original DPCC method by 1.03%, 1.25%, and 1.12% under the three operating conditions, respectively.

## 5. Conclusions

To address the issues of parameter sensitivity and poor robustness in continuous control set model predictive control strategies, this study combines model predictive control algorithms with the active disturbance rejection control algorithms to propose two improved model predictive control strategies. The resulting robust model predictive controller is then applied to PMSM.

First, through theoretical derivation, this study demonstrates the equivalence between the deadbeat current control based on an extended state observer and the active disturbance rejection control architecture, and calculates the errors introduced into the MPC algorithm’s predicted output by parameters such as motor resistance, flux linkage and inductance.

Second, a linear–nonlinear switching observer is employed to detect disturbances caused by parameter deviations in the system, and the observed disturbance values are fed back in real time to the CCS-MPCC. This optimized algorithm reduces current harmonics, minimizes torque ripple, lowers the system’s sensitivity to parameter variations, and enhances the system’s disturbance rejection capability under parameter mismatch conditions.

Finally, experimental validation of the proposed improved CCS-MPCC algorithm was conducted using a 5.5 kW permanent magnet synchronous motor and a physical motor test platform. The experimental results demonstrate that, under conditions of system parameter mismatch, the two improvement methods based on the linear–nonlinear switching observer can reduce stator current oscillations in the CCS-MPCC system, lower the system’s harmonic content, enhance disturbance rejection capability, and further reduce the parameter sensitivity of the MPC algorithm.

Future work will upgrade torque and current sensors for improved high-frequency measurement, and establish a closed-loop stability analysis framework by integrating Lyapunov and robust control theory to support engineering applications.

## 6. Challenges and Future Directions

The proposed method demonstrates satisfactory performance in simulations and limited experiments; however, the following shortcomings remain:

1. Constrained by the limited precision and sampling frequency of the sensors on our current experimental platform, high-frequency dynamic metrics such as torque ripple and current ripple cannot be fully quantified. Moreover, the current controller is based on an ARM Cortex-M3 core (without a hardware floating-point unit), which imposes constraints on computational capability. Future work will acquire high-precision torque and current sensors to reconstruct the measurement system, and will upgrade the control platform to a high-performance solution featuring a hardware floating-point unit or FPGA, thereby enabling more comprehensive performance evaluation and deployment of complex algorithms.
2. The closed-loop convergence, observer-error dynamics, and robustness of the switching process under parameter mismatch and measurement noise have not yet been fully resolved theoretically; future work will integrate Lyapunov theory, input-to-state stability (ISS) analysis, and robust control methods to establish a systematic stability analysis framework, thereby providing a more solid theoretical foundation for engineering applications.

## Figures and Tables

**Table 1:** The partial parameters of PMSM.

Motor Parameter	Value	Unit
Rated Power	5.5	kW
Rated Current	10.5	A
Rated Voltage	380	V
Permanent Magnet Flux Linkage	0.05	Wb
Stator Resistance	0.31	Ω
Stator Inductance	0.00173	H
Number of Pole Pairs	1	—
Moment of Inertia	164.33	kg·mm^2^

**Table 2:** Comparison of the THD values of the five algorithms under three working conditions.

Algorithm	Condition I THD (%)	Condition II THD (%)	Condition III THD (%)
DPCC	5.58	8.38	13.27
Improved LESO method	5.11	7.95	12.91
Improved NESO method	4.90	7.65	12.54
Improved LNS1 method	4.63	7.23	12.24
Improved LNS2 method	4.55	7.13	12.15

**Table 3:** The effect of the proposed algorithms on system performance under three working conditions.

Algorithm	Improvement Under Condition I (%)	Improvement Under Condition II (%)	Improvement Under Condition III (%)
Improved LESO method	0.47	0.43	0.36
Improved NESO method	0.68	0.73	0.73
Improved LNS1 method	0.95	1.15	1.02
Improved LNS2 method	1.03	1.25	1.12

## Data Availability

The original contributions presented in this study are included in the article. Further inquiries can be directed to the corresponding author.

## Appendix A

### Appendix A.1. Stability Verification of LNS1

Based on the mathematical models of PMSMs in the d–q axis of the rotor synchronous coordinate system as described in the literature, and referring to the motor’s stator voltage equation, stator flux linkage equation, electromagnetic torque equation, and mechanical motion equation [27], the following mathematical expressions can be obtained:

(A1) $${\dot{\omega }}_{m}=\frac{3p_{n}\psi_{d}}{2J}i_{q}+f_{m}$$

(A2) $${\dot{i}}_{q}=\frac{U_{q}}{L_{q}}+f_{q}$$

(A3) $${\dot{i}}_{d}=\frac{U_{d}}{L_{d}}+f_{d}$$

where

(A4) $$f_{m}=-\frac{T_{L}}{J}-\frac{B\omega_{m}}{J}$$

(A5) $$f_{q}=-\frac{R_{s}i_{q}}{L_{q}}-\frac{\omega_{r}L_{d}i_{d}+\omega_{r}\psi_{f}}{L_{q}}$$

(A6) $$f_{d}=-\frac{R_{s}i_{d}}{L_{d}}+\frac{\omega_{r}L_{q}i_{q}}{L_{d}}$$

Equations (A1)–(A3) are the canonical forms for designing ADRC. We give an example how to design ADRC in speed loop. Equation (A1) can be recast as

(A7) $$\left\{\begin{array}{c} {\dot{x}}_{1}=b_{0}\;\cdot \;i_{q}+x_{2}\\ {\dot{x}}_{2}=-h \end{array}\right.$$

where $x_{1}=\omega_{m}$, $b_{m}=\frac{3p_{n}\psi_{d}}{2J}$, $x_{2}=f_{m}+\left(b_{m}-b_{0}\right)i_{q}$ and $b_{0}$ is the estimated value of $b_{m}$.

Equation (19) adopts discontinuous switching, $\beta_{11}$, $\beta_{21}$, $\beta_{22}$, and $\beta_{23}$ are gain coefficients of ESO. The control law is $i_{q}^{ref}=\frac{u_{0}-z_{2}}{b_{0}}$ and $u_{0}=k_{11}\left(v_{1}-z_{1}\right)$, where $b_{0}$ is the estimated value of $b_{m}$ and $k_{11}$ is the control gain.

Define $e_{2}=z_{2}-x_{2}$, The error state Equations from (A7) and (18) are shown as follows:

(A8) $$\left\{\begin{array}{c} {\dot{e}}_{1}=e_{2}-\beta_{11}\;\cdot \;e_{1}\\ {\dot{e}}_{2}=h-F_{2}\;\cdot \;fal\left(e_{1},\alpha_{2},\delta_{1},\delta_{2}\right) \end{array}\right.$$

We present a theorem to show that the linear–nonlinear switching ESO in LNS1 is bounded-input-bounded-output (BIBO) stability.

If the parameters of system (A8) satisfy $0<\alpha_{2}=\frac{n}{m}<1$, $\beta_{11}>0$, $\beta_{21}>0$, $\beta_{22}>0$, $\beta_{23}>0$, 3n>m, $2m\beta_{22}=\left(m+n\right)\beta_{23}$, n>0, $|\delta_{2}{|}^{\frac{n}{m}-1}=\frac{\beta_{21}}{\beta_{23}}$, and $|h|<R_{1}$, where $R_{1}$ is positive number, then for any compact set *Q*, there exists a constant N>0 such that any solution *e* of the system (A8) with initial value e(0)∈Q, satisfies ||e(t)||≤N for all t≥0.

The candidate Lyapunov function V(e), constituting of multiple Lyapunov functions, of system (A8) can be proposed as

(A9) $$\left\{\begin{array}{cc} & V(e)=V_{13}\left(e\right)\;\;\left|e_{1}\right|\leq \delta_{1}\\ & V(e)=V_{12}\left(e\right)\;\;\delta_{1}<\left|e_{1}\right|\leq \delta_{2}\\ & V(e)=V_{11}\left(e\right)\;\;\left|e_{1}\right|>\delta_{2} \end{array}\right.$$

where

(A10) $$\left\{\begin{array}{c} V_{13}={\left(c_{3}e_{1}^{2}+be_{2}^{2}\right)}^{\frac{2m}{m+n}}+c_{3}e_{1}^{2}+be_{2}^{2}-ae_{1}e_{2}\\ V_{12}={\left({\left|e_{1}\right|}^{\frac{n}{m}+1}+be_{2}^{2}\right)}^{\frac{2m}{m+n}}+{\left|e_{1}\right|}^{\frac{n}{m}+1}+be_{2}^{2}-ae_{1}e_{2}\\ V_{11}={\left(c_{1}e_{1}^{2}+be_{2}^{2}\right)}^{\frac{2m}{m+n}}+c_{1}e_{1}^{2}+be_{2}^{2}-ae_{1}e_{2}. \end{array}\right.$$

The parameters of V(e) satisfy $b=\frac{1}{\beta_{23}}$, $c_{1}=\frac{\beta_{21}}{\beta_{23}}$, $c_{3}=\frac{1}{{\delta_{1}}^{\left(1-\alpha_{2}\right)}}$, $e={\left[e_{1},e_{2}\right]}^{T}$, and $a\in (0,R_{2})$ with $R_{2}=min\left\{R_{21},R_{22},R_{23},R_{24},R_{25},R_{26}\right\}$, where $R_{21}=\frac{2\beta_{11}\beta_{21}}{\beta_{21}\beta_{23}+\frac{1}{4}\beta_{11}^{2}\beta_{23}}$, $R_{22}=\frac{2\beta_{11}}{\beta_{23}+\frac{1}{4}\beta_{11}^{2}\delta_{1}^{\left(1-\alpha_{2}\right)}}$, $R_{23}=\frac{2\sqrt{\beta_{21}}}{\beta_{23}}$, $R_{24}=\frac{2}{\sqrt{\beta_{23}}}$, $R_{25}=\frac{2}{\sqrt{\beta_{23}{\delta_{1}}^{\left(1-\alpha_{2}\right)}}}$, $R_{26}=\frac{\left(m+n\right)\beta_{11}}{m\beta_{22}}$.

By mathematical deduction, $a\in (0,R_{2})$ can guarantee that (A11)–(A15) are true:

(A11) $$\left[\begin{array}{cc} a & -\frac{1}{2}a\beta_{11}\\ -\frac{1}{2}a\beta_{11} & \left(2\beta_{11}c_{1}-\beta_{21}a\right) \end{array}\right]≻0$$

(A12) $$\left[\begin{array}{cc} a & -\frac{a\beta_{11}}{2}\\ -\frac{a\beta_{11}}{2} & \left(-\frac{a\beta_{23}}{\delta_{1}^{1-\alpha_{2}}}+2c_{3}\beta_{11}\right) \end{array}\right]≻0$$

(A13) $$\left[\begin{array}{cc} c_{1} & -\frac{a}{2}\\ -\frac{a}{2} & b \end{array}\right]≻0$$

(A14) $$\left[\begin{array}{cc} 1 & -\frac{a}{2}\\ -\frac{a}{2} & b \end{array}\right]≻0$$

(A15) $$\left[\begin{array}{cc} c_{3} & -\frac{a}{2}\\ -\frac{a}{2} & b \end{array}\right]≻0$$

where ≻ represents the symbol for a positive definite matrix.

Step 1: By mathematical deduction, we can get $V_{11}\geq c_{1}e_{1}^{2}+be_{2}^{2}-ae_{1}e_{2}$, $V_{12}\geq {\left|e_{1}\right|}^{2}+be_{2}^{2}-ae_{1}e_{2}$, and $V_{13}\geq c_{3}e_{1}^{2}+be_{2}^{2}-ae_{1}e_{2}$, Inequalities (A13)–(A14) mean that $V_{1d}$ are positive definite and radially unbounded, where d∈{1,2,3}.

Step 2: We will show that the given functions do not increase during switching instants. And during continuous evolution, the function is decreasing. Computing the derivative of $V_{1d}$ along the solution of system (A8) yields

(A16) $$\begin{array}{cc} {\dot{V}}_{13}= & \frac{2m}{m+n}{\left(c_{3}e_{1}^{2}+be_{2}^{2}\right)}^{\frac{2m}{m+n}-1}\left[2c_{3}e_{1}e_{2}-2c_{3}\beta_{11}e_{1}^{2}+2be_{2}h-2b\frac{\beta_{23}e_{1}e_{2}}{\delta_{1}^{1-\alpha_{1}}}\right]+2c_{3}e_{1}e_{2}-2c_{3}\beta_{11}e_{1}^{2}+2be_{2}h\\ \; & -2b\frac{\beta_{23}e_{1}e_{2}}{\delta_{1}^{1-\alpha_{1}}}-ae_{2}^{2}+\beta_{11}ae_{2}e_{1}-ae_{1}h+\frac{\beta_{23}ae_{1}^{2}}{\delta_{1}^{1-\alpha_{1}}} \end{array}$$

(A17) $$\begin{array}{cc} {\dot{V}}_{12}= & \frac{2m}{m+n}{\left({\left|e_{1}\right|}^{\frac{n}{m}+1}+be_{2}^{2}\right)}^{\frac{m-n}{m+n}}\left[\frac{m+n}{m}{\left|e_{1}\right|}^{\frac{n}{m}}e_{2}sign\left(e_{1}\right)-\frac{m+n}{m}{\left|e_{1}\right|}^{\frac{n}{m}+1}\beta_{11}+2be_{2}h-2b\beta_{22}{\left|e_{1}\right|}^{\frac{n}{m}}e_{2}sign\left(e_{1}\right)\right]-\frac{m+n}{m}{\left|e_{1}\right|}^{\frac{n}{m}+1}\beta_{11}\\ \; & +\frac{m+n}{m}{\left|e_{1}\right|}^{\frac{n}{m}}e_{2}sign\left(e_{1}\right)+2be_{2}h-2be_{2}\beta_{22}{\left|e_{1}\right|}^{\frac{n}{m}}sign\left(e_{1}\right)-ae_{2}^{2}+\beta_{11}ae_{1}e_{2}-ae_{1}h+a\beta_{22}{\left|e_{1}\right|}^{\frac{n}{m}+1} \end{array}$$

(A18) $$\begin{array}{cc} {\dot{V}}_{11}= & \frac{2m}{m+n}{\left(c_{1}e_{1}^{2}+be_{2}^{2}\right)}^{\frac{2m}{m+n}-1}\left[2c_{1}e_{1}e_{2}-\beta_{11}2c_{1}e_{1}^{2}+2be_{2}h-\beta_{21}2be_{2}e_{1}\right]+[2c_{1}e_{1}e_{2}-\beta_{11}2c_{1}e_{1}^{2}+2be_{2}h\\ \; & -\beta_{21}2be_{2}e_{1}]-ae_{2}^{2}+\beta_{11}ae_{2}e_{1}-ae_{1}h+a\beta_{21}e_{1}^{2} \end{array}$$

The inequality (A11) results in $-ae_{2}^{2}+a\beta_{11}e_{1}e_{2}-\left(\beta_{11}2c_{1}-\beta_{21}a\right)e_{1}^{2}<0$. And when $c_{1}=\beta_{21}b$, (A18) can be recast as

(A19) $$\begin{array}{cc} {\dot{V}}_{11}= & -\beta_{11}2c_{1}e_{1}^{2}\frac{2m}{m+n}{\left(c_{1}e_{1}^{2}+be_{2}^{2}\right)}^{\frac{2m}{m+n}-1}-ae_{2}^{2}+\beta_{11}ae_{2}e_{1}-\left(2\beta_{11}c_{1}-a\beta_{21}\right)e_{1}^{2}\\ \; & +2be_{2}h\frac{2m}{m+n}{\left(c_{1}e_{1}^{2}+be_{2}^{2}\right)}^{\frac{2m}{m+n}-1}+2be_{2}h-ae_{1}h \end{array}$$

It is easy to derive the following inequality:

(A20) $${\left(c_{1}e_{1}^{2}+be_{2}^{2}\right)}^{\frac{2m}{m+n}-1}\leq {\left(2c_{1}e_{1}^{2}\right)}^{\frac{2m}{m+n}-1}+{\left(2be_{2}^{2}\right)}^{\frac{2m}{m+n}-1}$$

And then we have

(A21) $$e_{2}{\left(c_{1}e_{1}^{2}+be_{2}^{2}\right)}^{\frac{2m}{m+n}-1}\leq |e_{2}|{\left(2c_{1}e_{1}^{2}\right)}^{\frac{2m}{m+n}-1}+\left|e_{2}\right|{\left(2be_{2}^{2}\right)}^{\frac{2m}{m+n}-1}$$

Furthermore, the inequality (A21) can be recast as

(A22) $$e_{2}{\left(c_{1}e_{1}^{2}+be_{2}^{2}\right)}^{\frac{2m}{m+n}-1}\leq |e_{2}\left|(\right|e_{1}{\left|\right)}^{2\frac{2m}{m+n}-2}{\left(2c_{1}\right)}^{\frac{2m}{m+n}-1}+{\left|e_{2}\right|}^{2\frac{2m}{m+n}-1}{\left(2b\right)}^{\frac{2m}{m+n}-1}$$

By combining (A19) and inequality (A22), one can conclude that

(A23) $$\begin{array}{cc} {\dot{V}}_{11}\leq & -\beta_{11}2c_{1}e_{1}^{2}\frac{2m}{m+n}{\left(c_{1}e_{1}^{2}+be_{2}^{2}\right)}^{\frac{2m}{m+n}-1}-ae_{2}^{2}+\beta_{11}ae_{2}e_{1}-\left(2\beta_{11}c_{1}-a\beta_{21}\right)e_{1}^{2}+2be_{2}h-ae_{1}h\\ \; & +2bR_{1}\left[\right|e_{2}\left|(\right|e_{1}{\left|\right)}^{2\frac{2m}{m+n}-2}{\left(2c_{1}\right)}^{\frac{2m}{m+n}-1}+|e_{2}{|}^{2\frac{2m}{m+n}-1}{\left(2b\right)}^{\frac{2m}{m+n}-1}] \end{array}$$

The constraint m<3n results in $2\frac{2m}{m+n}-2<1$ and $2\frac{2m}{m+n}-1<2$. Toward that end, the order of the negative terms is larger than the order of the positive terms in (A25). Then with the constraint $0<\theta_{11}<1$, thus, the following result can be obtained:

(A24) $${\dot{V}}_{11}\leq -\left(1-\theta_{11}\right)\left[\beta_{11}2c_{1}e_{1}^{2}\frac{2m}{m+n}{\left(c_{1}e_{1}^{2}+be_{2}^{2}\right)}^{\frac{2m}{m+n}-1}+ae_{2}^{2}-\beta_{11}ae_{2}e_{1}+\left(2\beta_{11}c_{1}-a\beta_{21}\right)e_{1}^{2}\right]$$

when

(A25) $$\begin{array}{cc} g_{11}= & -\theta_{11}\left[\beta_{11}2c_{1}e_{1}^{2}\frac{2m}{m+n}{\left(c_{1}e_{1}^{2}+be_{2}^{2}\right)}^{\frac{2m}{m+n}-1}+ae_{2}^{2}-\beta_{11}ae_{2}e_{1}+\left(2\beta_{11}c_{1}-a\beta_{21}\right)e_{1}^{2}\right]\\ \; & +2bR_{1}\left[\right|e_{2}\left|(\right|e_{1}{\left|\right)}^{2\frac{2m}{m+n}-2}{\left(2c_{1}\right)}^{\frac{2m}{m+n}-1}+|e_{2}{|}^{2\frac{2m}{m+n}-1}{\left(2b\right)}^{\frac{2m}{m+n}-1}]+2be_{2}h-ae_{1}h \end{array}$$

And when $\frac{m+n}{m}=\beta_{22}2b$ and $\frac{m+n}{m\beta_{22}}\beta_{11}>a$, (A17) can be recast as

(A26) $$\begin{array}{cc} {\dot{V}}_{12}\leq & -2{\left({\left|e_{1}\right|}^{\frac{n}{m}+1}+be_{2}^{2}\right)}^{\frac{m-n}{m+n}}{\left|e_{1}\right|}^{\frac{n}{m}+1}\beta_{11}-ae_{2}^{2}+\frac{2m}{m+n}{\left({\left|e_{1}\right|}^{\frac{n}{m}+1}+be_{2}^{2}\right)}^{\frac{m-n}{m+n}}2be_{2}h\\ & +2be_{2}h+\beta_{11}ae_{1}e_{2}-ae_{1}h \end{array}$$

It is easy to verify the following inequality:

(A27) $$\left(\right|e_{1}{|}^{\frac{n}{m}+1}+be_{2}^{2}{)}^{\frac{m-n}{m+n}}\leq \left(2\right|e_{1}{|}^{\frac{n}{m}+1}{)}^{\frac{m-n}{m+n}}+{\left(2be_{2}^{2}\right)}^{\frac{m-n}{m+n}}=\left(\right|e_{1}{\left|\right)}^{\frac{m-n}{m}}2^{\frac{m-n}{m+n}}+{\left(e_{2}\right)}^{\frac{2(m-n)}{m+n}}{\left(2b\right)}^{\frac{m-n}{m+n}}$$

Thus, one can conclude that

(A28) $$e_{2}\left(\right|e_{1}{|}^{\frac{n}{m}+1}+be_{2}^{2}{)}^{\frac{m-n}{m+n}}\leq |e_{2}\left|\right|e_{1}{|}^{\frac{m-n}{m}}2^{\frac{m-n}{m+n}}+{\left|e_{2}\right|}^{\frac{2(m-n)}{m+n}+1}{\left(2b\right)}^{\frac{m-n}{m+n}}$$

With the constraints 3n>m and inequality (A28), the order of the negative terms is larger than the order of the positive terms in (A30). With $0<\theta_{12}<1$, we have

(A29) $${\dot{V}}_{12}\leq -\left(1-\theta_{12}\right)\left[2{\left({\left|e_{1}\right|}^{\frac{n}{m}+1}+be_{2}^{2}\right)}^{\frac{m-n}{m+n}}{\left|e_{1}\right|}^{\frac{n}{m}+1}\beta_{11}+ae_{2}^{2}\right]$$

when

(A30) $$\begin{array}{cc} g_{12}= & -\theta_{12}\left[2{\left({\left|e_{1}\right|}^{\frac{n}{m}+1}+be_{2}^{2}\right)}^{\frac{m-n}{m+n}}{\left|e_{1}\right|}^{\frac{n}{m}+1}\beta_{11}+ae_{2}^{2}\right]+\frac{2m}{m+n}{\left({\left|e_{1}\right|}^{\frac{n}{m}+1}+be_{2}^{2}\right)}^{\frac{m-n}{m+n}}2be_{2}h\\ & +2be_{2}h+\beta_{11}ae_{1}e_{2}-ae_{1}h\leq 0 \end{array}$$

With the constraints $c_{3}=\frac{b\beta_{23}}{\delta_{1}^{1-\alpha_{2}}}$ and inequality (A12), the following results can be obtained:

(A31) $$\begin{array}{cc} {\dot{V}}_{13}= & -2c_{3}\beta_{11}e_{1}^{2}\frac{2m}{m+n}{\left(c_{3}e_{1}^{2}+be_{2}^{2}\right)}^{\frac{2m}{m+n}-1}-ae_{2}^{2}+\beta_{11}ae_{2}e_{1}-\left(-\frac{\beta_{23}a}{\delta_{1}^{1-\alpha_{1}}}+2c_{3}\beta_{11}\right)e_{1}^{2}\\ & +2be_{2}h\frac{2m}{m+n}{\left(c_{3}e_{1}^{2}+be_{2}^{2}\right)}^{\frac{2m}{m+n}-1}-ae_{1}h+2be_{2}h \end{array}$$

There exists

(A32) $${\left(c_{3}e_{1}^{2}+be_{2}^{2}\right)}^{\frac{2m}{m+n}-1}\leq {\left(2c_{3}e_{1}^{2}\right)}^{\frac{2m}{m+n}-1}+{\left(2be_{2}^{2}\right)}^{\frac{2m}{m+n}-1}$$

One can conclude that

(A33) $$e_{2}{\left(c_{3}e_{1}^{2}+be_{2}^{2}\right)}^{\frac{2m}{m+n}-1}\leq |e_{2}|{\left(e_{1}\right)}^{2\frac{2m}{m+n}-2}{\left(2c_{3}\right)}^{\frac{2m}{m+n}-1}+\left(\right|e_{2}{\left|\right)}^{2\frac{2m}{m+n}-1}{\left(2b\right)}^{\frac{2m}{m+n}-1}$$

The constraint m<3n results in $2\frac{2m}{m+n}-2<1$ and $2\frac{2m}{m+n}-1<2$. Thus, the inequality (A33) can be recast as

(A34) $$e_{2}{\left(c_{3}e_{1}^{2}+be_{2}^{2}\right)}^{\frac{2m}{m+n}-1}<|e_{2}\left|\right|e_{1}|{\left(2c_{3}\right)}^{\frac{2m}{m+n}-1}+{\left|e_{2}\right|}^{2}{\left(2b\right)}^{\frac{2m}{m+n}-1}$$

Toward that end, the order of the negative terms is larger than the order of the positive terms in (A36). With $0<\theta_{13}<1$, it holds that

(A35) $${\dot{V}}_{13}\leq -\left(1-\theta_{13}\right)\left[2c_{3}\beta_{11}e_{1}^{2}\frac{2m}{m+n}{\left(c_{3}e_{1}^{2}+be_{2}^{2}\right)}^{\frac{2m}{m+n}-1}+ae_{2}^{2}-\beta_{11}ae_{2}e_{1}+\left(-\frac{\beta_{23}a}{\delta_{1}^{1-\alpha_{1}}}+2c_{3}\beta_{11}\right)e_{1}^{2}\right]$$

when

(A36) $$\begin{array}{cc} g_{13}= & -\theta_{13}\left[2c_{3}\beta_{11}e_{1}^{2}\frac{2m}{m+n}{\left(c_{3}e_{1}^{2}+be_{2}^{2}\right)}^{\frac{2m}{m+n}-1}+ae_{2}^{2}-\beta_{11}ae_{2}e_{1}+\left(-\frac{\beta_{23}a}{\delta_{1}^{1-\alpha_{1}}}+2c_{3}\beta_{11}\right)e_{1}^{2}\right]\\ \; & +2be_{2}h\frac{2m}{m+n}{\left(c_{3}e_{1}^{2}+be_{2}^{2}\right)}^{\frac{2m}{m+n}-1}-ae_{1}h+2be_{2}h\leq 0 \end{array}$$

At the switching points, $c_{1}={\left|\delta_{2}\right|}^{\frac{n}{m}-1}$ according to $|\delta_{2}{|}^{\frac{n}{m}-1}$ and $c_{1}=\frac{\beta_{21}}{\beta_{23}}$, and $c_{3}={\left|\delta_{1}\right|}^{\frac{n}{m}-1}$ according to $c_{3}=\frac{1}{{\delta_{1}}^{1-\alpha_{2}}}$ and $a_{2}=\frac{n}{m}$, can guarantee $V_{11}=V_{12}$ and $V_{13}=V_{12}$, respectively.

The detailed deduction is illustrated as follows:

When $|e_{1}|=\delta_{2}$, $c_{1}=\frac{\beta_{21}}{\beta_{23}}$ and $|\delta_{2}{|}^{\frac{n}{m}-1}=\frac{\beta_{21}}{\beta_{23}}$ can get $V_{11}=\left(\right|e_{1}{|}^{\frac{n}{m}+1}+be_{2}^{2}{)}^{\frac{2m}{m+n}}+{\left|e_{1}\right|}^{\frac{n}{m}+1}+be_{2}^{2}-ae_{1}e_{2}$. Then, we define $V_{12}=\left(\right|e_{1}{|}^{\frac{n}{m}+1}+be_{2}^{2}{)}^{\frac{2m}{m+n}}+{\left|e_{1}\right|}^{\frac{n}{m}+1}+be_{2}^{2}-ae_{1}e_{2}$. Thus, $V_{12}=V_{11}$.

When $|e_{1}|=\delta_{1}$, $c_{3}={\left|\delta_{1}\right|}^{\frac{n}{m}-1}$, we can obtain $V_{13}=\left(\right|e_{1}{|}^{\frac{n}{m}+1}+be_{2}^{2}{)}^{\frac{2m}{m+n}}+{\left|e_{1}\right|}^{\frac{n}{m}+1}+be_{2}^{2}-ae_{1}e_{2}$. $V_{12}=\left(\right|e_{1}{|}^{\frac{n}{m}+1}+be_{2}^{2}{)}^{\frac{2m}{m+n}}+{\left|e_{1}\right|}^{\frac{n}{m}+1}+be_{2}^{2}-ae_{1}e_{2}$ is defined by us, so we can get $V_{12}=V_{13}$.

When V(e)≥K for some K>0, $g_{11}\leq 0$, $g_{12}\leq 0$, $g_{13}\leq 0$, and thus, $\dot{V}\leq -\tilde{a}\left(|x|\right)$ where $\tilde{a}$ is a positive function. The stability proof of the LNS1 linear–nonlinear switching ESO has been completed.

### Appendix A.2. Stability Verification of LNS2

The following theorem shows that the linear–nonlinear switching ESO in LNS2 is BIBO stability.

Define $e_{2}=z_{2}-x_{2}$. The error state equations from (A7) and (20) are shown as follows:

(A37) $$\left\{\begin{array}{c} {\dot{e}}_{1}=e_{2}-F_{1}\;\cdot \;fal\left(e_{1},\alpha_{2},\delta_{1},\delta_{2}\right)\\ {\dot{e}}_{2}=h-F_{2}\;\cdot \;fal\left(e_{1},\alpha_{2},\delta_{1},\delta_{2}\right) \end{array}\right.$$

If the parameters of system (A37) satisfy $0<a_{2}=\frac{n}{m}<a_{1}=\frac{q}{p}<1$, $\beta_{i1}^{\left(1\right)}>0$, $\beta_{i1}^{\left(2\right)}>0$, $\beta_{i1}^{\left(3\right)}>0$, $2m\beta_{22}=\left(m+n\right)\beta_{23}$, 3n>m, $1<\frac{2n}{m}+\frac{q}{p}$, $|\delta_{2}{|}^{\frac{n}{m}-1}=\frac{\beta_{21}}{\beta_{23}}$, and $\left|h\right|\leq R_{1}$, where $R_{1}$ is positive number, then for any compact set *O*, there exists a constant M>0 such that any solution *e* of the system (A37) with initial value e(0)∈O, satisfies ||e(t)||≤M for all t≥0.

The candidate Lyapunov function V(e), constituting of multiple Lyapunov functions, of system (A37) is constructed as follows:

(A38) $$\left\{\begin{array}{cc} & V(e)=V_{23}\left(e\right)\;\;\left|e_{1}\right|\leq \delta_{1}\\ & V(e)=V_{22}\left(e\right)\;\;\delta_{1}<\left|e_{1}\right|\leq \delta_{2}\\ & V(e)=V_{21}\left(e\right)\;\;\left|e_{1}\right|>\delta_{2} \end{array}\right.$$

where

(A39) $$\left\{\begin{array}{cc} & V_{23}={\left(c_{3}e_{1}^{2}+be_{2}^{2}\right)}^{\frac{2m}{\left(m+n\right)}}+c_{3}e_{1}^{2}+be_{2}^{2}-ae_{1}e_{2}\\ & V_{22}=\left(\right|e_{1}{|}^{\frac{n}{m}+1}+be_{2}^{2}{)}^{\frac{2m}{\left(m+n\right)}}+{\left|e_{1}\right|}^{\frac{n}{m}+1}+be_{2}^{2}-ae_{1}e_{2}\\ & V_{21}={\left(c_{1}e_{1}^{2}+be_{2}^{2}\right)}^{\frac{2m}{\left(m+n\right)}}+c_{1}e_{1}^{2}+be_{2}^{2}-ae_{1}e_{2}. \end{array}\right.$$

The parameters of V(e) satisfy $b=\frac{1}{\beta_{23}}$, $c_{1}=\frac{\beta_{21}}{\beta_{23}}$, $c_{3}=\frac{1}{{\delta_{1}}^{\left(1-\alpha_{2}\right)}}$, $e={\left[e_{1},e_{2}\right]}^{T}$, and $a\in (0,R_{3})$ with $R_{3}=min\left\{R_{31},R_{32},R_{33},R_{34},R_{35}\right\}$, where $R_{31}=\frac{2\beta_{11}^{\left(1\right)}\beta_{21}}{\beta_{21}\beta_{23}+\frac{1}{4}{\left[\beta_{11}^{\left(1\right)}\right]}^{2}\beta_{23}}$, $R_{32}=\frac{8\beta_{11}^{\left(3\right)}{\delta_{1}}^{\left(\alpha_{1}-\alpha_{2}\right)}}{4\beta_{23}{\delta_{1}}^{\left(1-\alpha_{2}\right)}+{\left[\beta_{11}^{\left(3\right)}\right]}^{2}}$, $R_{33}=\frac{2\sqrt{\beta_{21}}}{\beta_{23}}$, $R_{34}=\frac{2}{\sqrt{\beta_{23}}}$, $R_{35}=\frac{2}{\sqrt{\beta_{23}{\delta_{1}}^{\left(1-\alpha_{2}\right)}}}$.

By mathematical deduction, $a\in (0,R_{3})$ can guarantee that the following inequalities hold:

(A40) $$\left[\begin{array}{cc} a & -\frac{1}{2}a\beta_{11}^{\left(1\right)}\\ -\frac{1}{2}a\beta_{11}^{\left(1\right)} & \left(2\beta_{11}^{\left(1\right)}c_{1}-\beta_{21}a\right) \end{array}\right]≻0$$

(A41) $$\left[\begin{array}{cc} a & -\frac{a\beta_{11}^{\left(3\right)}}{2\delta_{1}^{1-\alpha_{1}}}\\ -\frac{a\beta_{11}^{\left(3\right)}}{2\delta_{1}^{1-\alpha_{1}}} & \left(-\frac{a\beta_{23}}{\delta_{1}^{1-\alpha_{2}}}+\frac{2c_{3}\beta_{11}^{\left(3\right)}}{\delta_{1}^{1-\alpha_{1}}}\right) \end{array}\right]≻0$$

(A42) $$\left[\begin{array}{cc} c_{1} & -\frac{a}{2}\\ -\frac{a}{2} & b \end{array}\right]≻0$$

(A43) $$\left[\begin{array}{cc} 1 & -\frac{a}{2}\\ -\frac{a}{2} & b \end{array}\right]≻0$$

(A44) $$\left[\begin{array}{cc} c_{3} & -\frac{a}{2}\\ -\frac{a}{2} & b \end{array}\right]≻0$$

Step 1: By mathematical deduction, we can get $V_{21}\geq c_{1}e_{1}^{2}+be_{2}^{2}-ae_{1}e_{2}$, $V_{22}\geq {\left|e_{1}\right|}^{2}+be_{2}^{2}-ae_{1}e_{2}$, and $V_{23}\geq c_{3}e_{1}^{2}+be_{2}^{2}-ae_{1}e_{2}$, Inequalities (A42)–(A44) mean that $V_{2d}$ are positive definite and radially unbounded, where d∈{1,2,3}.

Step 2: We will show that the given functions do not increase during switching instants. And during continuous evolution, the function is decreasing.

Computing the derivative of $V_{2d}$ along the solution of system (A37) yields

(A45) $$\begin{array}{cc} {\dot{V}}_{23}= & \frac{2m}{\left(m+n\right)}{\left(c_{3}e_{1}^{2}+be_{2}^{2}\right)}^{\frac{2m}{\left(m+n\right)}-1}\left[2c_{3}e_{1}e_{2}-2c_{3}\beta_{11}^{\left(3\right)}\frac{e_{1}^{2}}{\delta_{1}^{1-\alpha_{1}}}+2be_{2}h-2b\beta_{23}\frac{e_{1}e_{2}}{\delta_{1}^{1-\alpha_{2}}}\right]+[2c_{3}e_{1}e_{2}-2c_{3}\beta_{11}^{\left(3\right)}\frac{e_{1}^{2}}{\delta_{1}^{1-\alpha_{1}}}\\ \; & +2be_{2}h-2b_{3}\beta_{23}\frac{e_{1}e_{2}}{\delta_{1}^{1-\alpha_{2}}}]-ae_{2}e_{2}+a\beta_{11}^{\left(3\right)}\frac{e_{1}e_{2}}{\delta_{1}^{1-\alpha_{1}}}-ae_{1}h+a\beta_{23}\frac{e_{1}^{2}}{\delta_{1}^{1-\alpha_{2}}} \end{array}$$

(A46) $$\begin{array}{cc} {\dot{V}}_{22}= & \frac{2m}{\left(m+n\right)}{\left({\left|e_{1}\right|}^{\frac{n}{m}+1}+be_{2}^{2}\right)}^{\frac{2m}{\left(m+n\right)}-1}\times \{\frac{m+n}{m}{\left|e_{1}\right|}^{\frac{n}{m}}e_{2}sign\left(e_{1}\right)-\beta_{11}^{\left(2\right)}{\left|e_{1}\right|}^{\frac{q}{p}}\frac{m+n}{m}{\left|e_{1}\right|}^{\frac{n}{m}}+2be_{2}h-\beta_{22}2be_{2}{\left|e_{1}\right|}^{\frac{n}{m}}sign\left(e_{1}\right)\}\\ \; & +\{\frac{m+n}{m}{\left|e_{1}\right|}^{\frac{n}{m}}e_{2}sign\left(e_{1}\right)-\frac{m+n}{m}\beta_{11}^{\left(2\right)}{\left|e_{1}\right|}^{\frac{n}{m}+\frac{q}{p}}+2be_{2}h-\beta_{22}2be_{2}{\left|e_{1}\right|}^{\frac{n}{m}}sign\left(e_{1}\right)\}-ae_{2}^{2}+\beta_{22}a{\left|e_{1}\right|}^{\frac{n}{m}+1}\\ \; & +a\beta_{11}^{\left(2\right)}{\left|e_{1}\right|}^{\frac{q}{p}}e_{2}sign\left(e_{1}\right)-ae_{1}h \end{array}$$

(A47) $$\begin{array}{cc} {\dot{V}}_{21}= & \frac{2m}{\left(m+n\right)}{\left(c_{1}e_{1}^{2}+be_{2}^{2}\right)}^{\frac{2m}{\left(m+n\right)}-1}\left[\left(2c_{1}e_{1}e_{2}-2\beta_{11}^{\left(1\right)}c_{1}e_{1}^{2}\right)+\left(2be_{2}h-2\beta_{21}be_{2}e_{1}\right)\right]+[\left(2c_{1}e_{1}e_{2}-2\beta_{11}^{\left(1\right)}c_{1}e_{1}^{2}\right)\\ \; & +\left(2be_{2}h-2\beta_{21}be_{2}e_{1}\right)]-ae_{2}^{2}+a\beta_{11}^{\left(1\right)}e_{1}e_{2}-ae_{1}h+\beta_{21}ae_{1}^{2} \end{array}$$

The inequality (A40) results in $-ae_{2}^{2}+\beta_{11}^{\left(1\right)}ae_{2}e_{1}-\left(\beta_{11}^{\left(1\right)}2c_{1}-\beta_{21}a\right)e_{1}^{2}<0$. And when $c_{1}=\beta_{21}b$, (A47) can be recast as

(A48) $$\begin{array}{cc} {\dot{V}}_{21}= & \frac{2m}{\left(m+n\right)}{\left(c_{1}e_{1}^{2}+be_{2}^{2}\right)}^{\frac{2m}{\left(m+n\right)}-1}\left[-2\beta_{11}^{\left(1\right)}c_{1}e_{1}^{2}+2be_{2}h\right]+2be_{2}h-ae_{1}h-ae_{2}^{2}+a\beta_{11}^{\left(1\right)}e_{1}e_{2}-\left(2\beta_{11}^{\left(1\right)}c_{1}-\beta_{21}a\right)e_{1}^{2} \end{array}$$

It is easy to derive

(A49) $$2{\left(c_{1}e_{1}^{2}+be_{2}^{2}\right)}^{\frac{2m}{\left(m+n\right)}-1}leq|e_{2}|{\left(2c_{1}e_{1}^{2}\right)}^{\frac{2m}{\left(m+n\right)}-1}+\left|e_{2}\right|{\left(2be_{2}^{2}\right)}^{\frac{2m}{\left(m+n\right)}-1}$$

Furthermore, we can obtain

(A50) $$e_{2}{\left(c_{1}e_{1}^{2}+be_{2}^{2}\right)}^{\frac{2m}{\left(m+n\right)}-1}\leq |e_{2}\left|\right|e_{1}{|}^{\frac{2(m-n)}{m+n}}{\left(2c_{1}\right)}^{\frac{m-n}{m+n}}+{\left|e_{2}\right|}^{\frac{3m-n}{m+n}}{\left(2b\right)}^{\frac{m-n}{m+n}}$$

Then the constraint m<3n results in $\frac{1}{2}>\frac{2m}{m+n}-1>0$ and $3>\frac{4m}{m+n}$, and $0<\theta_{21}<1$ and the inequality (A50), the order of the negative terms is larger than the order of the positive terms in (A52). Thus, the following result can be obtained:

(A51) $${\dot{V}}_{21}\leq -\left(1-\theta_{21}\right)\left[2\beta_{11}^{\left(1\right)}c_{1}e_{1}^{2}\frac{2m}{m+n}{\left(c_{1}e_{1}^{2}+be_{2}^{2}\right)}^{\frac{m-n}{m+n}}+ae_{2}^{2}-a\beta_{11}^{\left(1\right)}e_{1}e_{2}+\left(2\beta_{11}^{\left(1\right)}c_{1}-\beta_{21}a\right)e_{1}^{2}\right]$$

when

(A52) $$\begin{array}{cc} g_{21}= & -\theta_{21}\left[2\beta_{11}^{\left(1\right)}c_{1}e_{1}^{2}\frac{2m}{m+n}{\left(c_{1}e_{1}^{2}+be_{2}^{2}\right)}^{\frac{m-n}{m+n}}+ae_{2}^{2}-a\beta_{11}^{\left(1\right)}e_{1}e_{2}+\left(2\beta_{11}^{\left(1\right)}c_{1}-\beta_{21}a\right)e_{1}^{2}\right]\\ & +2be_{2}h\frac{2m}{m+n}{\left(c_{1}e_{1}^{2}+be_{2}^{2}\right)}^{\frac{m-n}{m+n}}+2be_{2}h-ae_{1}h\leq 0 \end{array}$$

when $\frac{m+n}{m}=2\beta_{22}b$, (A46) can be recast as

(A53) $$\begin{array}{cc} {\dot{V}}_{22}= & -\beta_{11}^{\left(2\right)}2{\left({\left|e_{1}\right|}^{\frac{n}{m}+1}+be_{2}^{2}\right)}^{\frac{2m}{\left(m+n\right)}-1}{\left|e_{1}\right|}^{\frac{n}{m}+\frac{q}{p}}+\frac{2m}{\left(m+n\right)}{\left({\left|e_{1}\right|}^{\frac{n}{m}+1}+be_{2}^{2}\right)}^{\frac{2m}{\left(m+n\right)}-1}2be_{2}h-\frac{m+n}{m}\beta_{11}^{\left(2\right)}{\left|e_{1}\right|}^{\frac{n}{m}+\frac{q}{p}}\\ & +2be_{2}h-ae_{2}^{2}+a\beta_{11}^{\left(2\right)}{\left|e_{1}\right|}^{\frac{q}{p}}e_{2}sign\left(e_{1}\right)-ae_{1}h+\beta_{22}a{\left|e_{1}\right|}^{\frac{n}{m}+1} \end{array}$$

Using Young’s inequality leads to $|a\beta_{11}^{\left(2\right)}|e_{1}{|}^{\frac{q}{p}}e_{2}sign\left(e_{1}\right)|\leq \frac{1}{2}k_{1}|a\beta_{11}^{\left(2\right)}\left|\right|e_{1}{|}^{2\frac{q}{p}}+\frac{1}{2k_{1}}\left|a\beta_{11}^{\left(2\right)}\right|$$e_{2}^{2}$, with $k_{1}>0$. One can conclude that

(A54) $$\begin{array}{cc} {\dot{V}}_{22}\leq & -\beta_{11}^{\left(2\right)}2{\left({\left|e_{1}\right|}^{\frac{n}{m}+1}+be_{2}^{2}\right)}^{\frac{2m}{\left(m+n\right)}-1}{\left|e_{1}\right|}^{\frac{n}{m}+\frac{q}{p}}+\frac{2m}{\left(m+n\right)}{\left({\left|e_{1}\right|}^{\frac{n}{m}+1}+be_{2}^{2}\right)}^{\frac{2m}{\left(m+n\right)}-1}2be_{2}h-\frac{m+n}{m}\beta_{11}^{\left(2\right)}{\left|e_{1}\right|}^{\frac{n}{m}+\frac{q}{p}}\\ & +2be_{2}h-ae_{2}^{2}+\frac{1}{2}k_{1}\left|a\beta_{11}^{\left(2\right)}\right|{\left|e_{1}\right|}^{2\frac{q}{p}}+\frac{1}{2k_{1}}\left|a\beta_{11}^{\left(2\right)}\right|e_{2}^{2}-ae_{1}h+\beta_{22}a{\left|e_{1}\right|}^{\frac{n}{m}+1} \end{array}$$

With

(A55) $${\left({\left|e_{1}\right|}^{\frac{n}{m}+1}+be_{2}^{2}\right)}^{\frac{2m}{\left(m+n\right)}-1}\leq {\left(2{\left|e_{1}\right|}^{\frac{n}{m}+1}\right)}^{\frac{2m}{\left(m+n\right)}-1}+{\left(2be_{2}^{2}\right)}^{\frac{2m}{\left(m+n\right)}-1}$$

We can obtain

(A56) $$\begin{array}{cc} {\dot{V}}_{22}\leq & -\beta_{11}^{\left(2\right)}2{\left({\left|e_{1}\right|}^{\frac{n}{m}+1}+be_{2}^{2}\right)}^{\frac{2m}{\left(m+n\right)}-1}{\left|e_{1}\right|}^{\frac{n}{m}+\frac{q}{p}}+\left[\frac{2m}{\left(m+n\right)}{\left(2{\left|e_{1}\right|}^{\frac{n}{m}+1}\right)}^{\frac{2m}{\left(m+n\right)}-1}2be_{2}h+\frac{2m}{\left(m+n\right)}{\left(be_{2}^{2}\right)}^{\frac{2m}{\left(m+n\right)}-1}2be_{2}h\right]\\ & -\frac{m+n}{m}\beta_{11}^{\left(2\right)}{\left|e_{1}\right|}^{\frac{n}{m}+\frac{q}{p}}+2be_{2}h-ae_{2}^{2}+\frac{1}{2}k_{1}\left|a\beta_{11}^{\left(2\right)}\right|{\left|e_{1}\right|}^{2\frac{q}{p}}+\frac{1}{2k_{1}}\left|a\beta_{11}^{\left(2\right)}\right|e_{2}^{2}-ae_{1}h+\beta_{22}a{\left|e_{1}\right|}^{\frac{n}{m}+1} \end{array}$$

Using Young’s inequality, we have $\frac{2hmb}{m+n}2(2|e_{1}{|}^{\frac{n}{m}\;+\;1}{)}^{\frac{2m}{m+n}\;-\;1}e_{2}\leq \frac{2hmb}{m+n}k_{2}\left[2(2\right|e_{1}{|}^{\frac{n}{m}\;+\;1}{{)}^{\frac{2m}{m+n}\;-\;1}]}^{2}$$+\frac{2hmb}{m+n}\frac{1}{k_{2}}e_{2}^{2}$, which satisfies $k_{2}>0$. Because $\frac{2n}{m}+\frac{q}{p}>1$, we have $2\left(\frac{n}{m}+1\right)\left(\frac{2m}{m+n}-1\right)<\left(\frac{n}{m}+1\right)\left(\frac{2m}{m+n}-1\right)+\frac{n}{m}+\frac{q}{p}$, And $0<\theta_{22}<1$.

Then the following results can be obtained:

(A57) $$\begin{array}{cc} {\dot{V}}_{22}\leq & -\left(1-\theta_{22}\right)\left\{\beta_{11}^{\left(2\right)}2{\left({\left|e_{1}\right|}^{\frac{n}{m}+1}+be_{2}^{2}\right)}^{\frac{2m}{\left(m+n\right)}-1}{\left|e_{1}\right|}^{\frac{n}{m}+\frac{q}{p}}+\frac{m+n}{m}\beta_{11}^{\left(2\right)}{\left|e_{1}\right|}^{\frac{n}{m}+\frac{q}{p}}+ae_{2}^{2}\right\}-\theta_{22}\{\beta_{11}^{\left(2\right)}2{\left({\left|e_{1}\right|}^{\frac{n}{m}+1}+be_{2}^{2}\right)}^{\frac{2m}{\left(m+n\right)}-1}{\left|e_{1}\right|}^{\frac{n}{m}+\frac{q}{p}}\\ & +\frac{m+n}{m}\beta_{11}^{\left(2\right)}{\left|e_{1}\right|}^{\frac{n}{m}+\frac{q}{p}}+ae_{2}^{2}\}+\left[\frac{2mbh}{\left(m+n\right)}k_{2}{\left[2{\left(2{\left|e_{1}\right|}^{\frac{n}{m}+1}\right)}^{\frac{2m}{m+n}-1}\right]}^{2}+\frac{2mbh}{\left(m+n\right)}\frac{1}{k_{2}}e_{2}^{2}+\frac{2m}{\left(m+n\right)}{\left(be_{2}^{2}\right)}^{\frac{2m}{\left(m+n\right)}-1}2be_{2}h\right]\\ & +2be_{2}h+\frac{1}{2}k_{1}\left|a\beta_{11}^{\left(2\right)}\right|{\left|e_{1}\right|}^{2\frac{q}{p}}+\frac{1}{2k_{1}}\left|a\beta_{11}^{\left(2\right)}\right|e_{2}^{2}-ae_{1}h+\beta_{22}a{\left|e_{1}\right|}^{\frac{n}{m}+1} \end{array}$$

The inequality (A57) can be recast as

(A58) $${\dot{V}}_{22}\leq -\left(1-\theta_{22}\right)\left\{\beta_{11}^{\left(2\right)}2{\left({\left|e_{1}\right|}^{\frac{n}{m}+1}+be_{2}^{2}\right)}^{\frac{2m}{\left(m+n\right)}-1}{\left|e_{1}\right|}^{\frac{n}{m}+\frac{q}{p}}+\frac{m+n}{m}\beta_{11}^{\left(2\right)}{\left|e_{1}\right|}^{\frac{n}{m}+\frac{q}{p}}+ae_{2}^{2}\right\}$$

when

(A59) $$\begin{array}{cc} g_{22}= & -\theta_{22}\left\{\beta_{11}^{\left(2\right)}2{\left({\left|e_{1}\right|}^{\frac{n}{m}+1}+be_{2}^{2}\right)}^{\frac{2m}{\left(m+n\right)}-1}{\left|e_{1}\right|}^{\frac{n}{m}+\frac{q}{p}}+\frac{m+n}{m}\beta_{11}^{\left(2\right)}{\left|e_{1}\right|}^{\frac{n}{m}+\frac{q}{p}}+ae_{2}^{2}\right\}+\{\frac{2mbh}{\left(m+n\right)}k_{2}{\left[2{\left(2{\left|e_{1}\right|}^{\frac{n}{m}+1}\right)}^{\frac{2m}{\left(m+n\right)}-1}\right]}^{2}\\ & +\frac{2mbh}{\left(m+n\right)}\frac{1}{k_{2}}e_{2}^{2}+\frac{2m}{\left(m+n\right)}{\left(be_{2}^{2}\right)}^{\frac{2m}{\left(m+n\right)}-1}2be_{2}h\}+2be_{2}h+\frac{1}{2}k_{1}\left|a\beta_{11}^{\left(2\right)}\right|{\left|e_{1}\right|}^{2\frac{q}{p}}+\frac{1}{2k_{1}}\left|a\beta_{11}^{\left(2\right)}\right|e_{2}^{2}-ae_{1}h\\ & +\beta_{22}a{\left|e_{1}\right|}^{\frac{n}{m}+1}\leq 0 \end{array}$$

Furthermore, with the constraints $3>2\frac{2m}{m+n}$, $\frac{n}{m}+1<2$, $2\frac{q}{p}<2$, and $2\left(\frac{n}{m}+1\right)\left(\frac{2m}{m+n}-1\right)<\left(\frac{n}{m}+1\right)\left(\frac{2m}{m+n}-1\right)+\frac{n}{m}+\frac{q}{p}$ aforementioned, we can conclude that the order of the negative terms is larger than the order of the positive terms in (A59).

With the constraints $c_{3}=\frac{b\beta_{23}}{{\delta_{1}}^{1-\alpha_{2}}}$ and inequality (A41), the following results can be obtained from (A45):

(A60) $$\begin{array}{cc} {\dot{V}}_{23}= & -\frac{2m}{\left(m+n\right)}2c_{3}\beta_{11}^{\left(3\right)}\frac{e_{1}^{2}}{\delta_{1}^{1-\alpha_{1}}}{\left(c_{3}e_{1}^{2}+be_{2}^{2}\right)}^{\frac{2m}{\left(m+n\right)}-1}-ae_{2}^{2}+a\beta_{11}^{\left(3\right)}\frac{e_{1}e_{2}}{\delta_{1}^{1-\alpha_{1}}}-\left(-\frac{a\beta_{23}}{\delta_{1}^{1-\alpha_{2}}}+\frac{2c_{3}\beta_{11}^{\left(3\right)}}{\delta_{1}^{1-\alpha_{1}}}\right)e_{1}^{2}\\ & +2be_{2}h\frac{2m}{\left(m+n\right)}{\left(c_{3}e_{1}^{2}+be_{2}^{2}\right)}^{\frac{2m}{\left(m+n\right)}-1}+2be_{2}h-ae_{1}h \end{array}$$

We can derive the following inequality according to Young’s inequality:

(A61) $$e_{2}{\left(c_{3}e_{1}^{2}+be_{2}^{2}\right)}^{\frac{2m}{\left(m+n\right)}-1}\leq |e_{2}|{\left(2c_{3}e_{1}^{2}\right)}^{\frac{2m}{\left(m+n\right)}-1}+\left|e_{2}\right|{\left(2be_{2}^{2}\right)}^{\frac{2m}{\left(m+n\right)}-1}$$

Furthermore, we can obtain

(A62) $$e_{2}{\left(c_{3}e_{1}^{2}+be_{2}^{2}\right)}^{\frac{2m}{\left(m+n\right)}-1}\leq |e_{2}\left|\right|e_{1}{|}^{\frac{2(m-n)}{m+n}}{\left(2c_{3}\right)}^{\frac{2m}{\left(m+n\right)}-1}+{\left|e_{2}\right|}^{\frac{3m-n}{m+n}}{\left(2b\right)}^{\frac{2m}{\left(m+n\right)}-1}$$

Then the constraint m<3n results in $\frac{1}{2}>\frac{2m}{m+n}-1>0$ and $3>\frac{4m}{m+n}$. Moreover, with the constraints $0<\theta_{23}<1$ and the inequality (A62), we can conclude that the order of the negative terms is larger than the order of the positive terms in (A64). Moreover, the sequel results can be presented as

(A63) $${\dot{V}}_{23}\leq -\left(1-\theta_{23}\right)\left[\frac{2m}{\left(m+n\right)}2c_{3}\beta_{11}^{\left(3\right)}\frac{e_{1}^{2}}{\delta_{1}^{1-\alpha_{1}}}{\left(c_{3}e_{1}^{2}+be_{2}^{2}\right)}^{\frac{2m}{\left(m+n\right)}-1}+ae_{2}^{2}\;-\;a\beta_{11}^{\left(3\right)}\frac{e_{1}e_{2}}{\delta_{1}^{1-\alpha_{1}}}\;+\;\left(\;-\;\frac{a\beta_{23}}{\delta_{1}^{1\;-\;\alpha_{2}}}\;+\;\frac{2c_{3}\beta_{11}^{\left(3\right)}}{\delta_{1}^{1-\alpha_{1}}}\right)e_{1}^{2}\right]$$

when

(A64) $$\begin{array}{cc} g_{23}= & -\theta_{23}\left[\frac{2m}{\left(m+n\right)}2c_{3}\beta_{11}^{\left(3\right)}\frac{e_{1}^{2}}{\delta_{1}^{1-\alpha_{1}}}{\left(c_{3}e_{1}^{2}+be_{2}^{2}\right)}^{\frac{2m}{\left(m+n\right)}-1}+ae_{2}^{2}-a\beta_{11}^{\left(3\right)}\frac{e_{1}e_{2}}{\delta_{1}^{1-\alpha_{1}}}+\left(-\frac{a\beta_{23}}{\delta_{1}^{1-\alpha_{2}}}+\frac{2c_{3}\beta_{11}^{\left(3\right)}}{\delta_{1}^{1-\alpha_{1}}}\right)e_{1}^{2}\right]\\ & +2be_{2}h\frac{2m}{\left(m+n\right)}{\left(c_{3}e_{1}^{2}+be_{2}^{2}\right)}^{\frac{2m}{\left(m+n\right)}-1}+2be_{2}h-ae_{1}h\leq 0 \end{array}$$

At the switching points, $c_{1}={\left|\delta_{2}\right|}^{\frac{n}{m}-1}$ according to $|\delta_{2}{|}^{\frac{n}{m}-1}$ and $c_{1}=\frac{\beta_{21}}{\beta_{23}}$, and $c_{3}={\left|\delta_{1}\right|}^{\frac{n}{m}-1}$ according to $c_{3}=\frac{1}{{\delta_{1}}^{1-\alpha_{2}}}$ and $\alpha_{2}=\frac{n}{m}$, can guarantee $V_{21}=V_{22}$ and $V_{23}=V_{22}$, respectively.

When V(e)≥K for some K>0, $g_{21}\leq 0$, $g_{22}\leq 0$, $g_{23}\leq 0$, and thus, $\dot{V}\leq -\tilde{a}\left(|x|\right)$ where $\tilde{a}$ is a positive function. The stability proof of the LNS2 linear–nonlinear switching ESO has been completed.
